# HCN4 gain-of-function mutation increases intrinsic heart rate and limits maladaptive remodeling under pressure overload

**DOI:** 10.3389/fphar.2026.1840832

**Published:** 2026-06-10

**Authors:** Konstantin Hennis, Julia Rilling, Linh Pham, René Rötzer, Chiara Piantoni, Yakun Wu, Nicolas Auerbach, Jürgen Groher, Daniela Kruck, Stefan Koplitz-Weißgerber, Christian Gruner, Andreas Scharr, Verena Mehlfeld, Martin Biel, Christian Wahl-Schott, Stefanie Fenske

**Affiliations:** 1 Department of Pharmacy – Center for Drug Research, Pharmacology for Natural Sciences, Ludwig-Maximilians Universität München, Munich, Germany; 2 Institute of Cardiovascular Physiology and Pathophysiology, Biomedical Center Munich, Ludwig-Maximilians Universität München, Planegg-Martinsried, Germany

**Keywords:** adaptive remodeling, gain-of-function mutation, HCN4, pacemaker channels, pressure-overload, sinoatrial node, sinus tachycardia

## Abstract

**Introduction:**

Gain-of-function (GOF) mutations in the cardiac pacemaker channel HCN4 have been associated with inappropriate sinus tachycardia in human patients. Chronic tachycardia is generally associated with adverse cardiac remodeling and cardiomyopathy, but whether enhanced HCN4 activity can induce or modulate such remodeling remains unknown. We aimed to investigate the influence of an HCN4 gain-of-function mutation on cardiac function and structure under baseline and pressure overload conditions.

**Methods and Results:**

We generated HCN4(Y527F) knock-in mice (HCN4F) carrying a GOF mutation in the C-linker of HCN4 channels, which shifts their activation curves to more positive potentials. Electrophysiological recordings confirmed increased channel availability at physiological membrane potentials. Telemetric ECGs and *in vivo* electrophysiological studies revealed an elevated mean and intrinsic heart rate, faster sinus node and atrioventricular conduction in HCN4F mice, but no spontaneous arrhythmias. In HCN4F mice, the heart rate histogram was truncated at lower heart rates, indicating fewer low-rate intervals and more frequent periods of elevated heart rate, while maximal heart rates remained comparable between the two phenotypes. Histological analysis did not reveal structural changes consistent with tachycardia-induced cardiomyopathy. Cardiac morphology, fibrosis, and contractility were indistinguishable between genotypes up to 12 months of age. Following transverse aortic constriction, both WT and HCN4F mice developed left ventricular hypertrophy, but HCN4F hearts exhibited less chamber dilatation, smaller left ventricular lumen, and preserved systolic function compared to WT. Gene expression and RNA-sequencing analyses revealed activation of a typical hypertrophic gene program in both genotypes, but distinct remodeling signatures.

**Discussion:**

The HCN4(Y527F) gain-of-function mutation increases intrinsic heart rate without inducing structural or functional deterioration. Under pressure overload, it even confers considerable protection against maladaptive dilatation and contractile dysfunction. These findings challenge the concept that persistent inappropriately elevated heart rate is necessarily detrimental and suggest that enhanced HCN4 activity may facilitate adaptive cardiac responses to stress.

## 1 Introduction

Hyperpolarization-activated cyclic nucleotide-gated (HCN) channels are key determinants of cardiac pacemaker activity. Among the four isoforms expressed in mammals, HCN4 is the predominant isoform in the sinoatrial node (SAN) of the heart, where it contributes to the funny current (I_f_) that drives diastolic depolarization and hence the generation of spontaneous pacemaker action potentials (APs) (Hennis et al., 2022a; Brown and Difrancesco, 1980; Biel et al., 2009). Loss-of-function mutations in HCN4 have been linked to a wide spectrum of cardiac disorders, including sinus node dysfunction and inherited bradycardia (for review see (Hennis et al., 2024)). But also more complex HCN4-associated arrhythmias have been described, including prolonged QT (Ueda et al., 2004), AV-Block (Zhou et al., 2014), Brugada syndrome (Ueda et al., 2009), early onset atrial fibrillation (Macri et al., 2014), and bradycardia-tachycardia syndrome (Duhme et al., 2013). Moreover, structural diseases like left-ventricular non-compaction cardiomyopathy have been linked to HCN channels (Milano et al., 2014; Yokoyama et al., 2018). Recently, gain-of-function mutations in HCN4 have been identified in patients. Such GOF mutations increase HCN4 channel availability by shifting activation to more positive potentials, thereby increasing heart rate manifesting clinically as inappropriate sinus tachycardia (IST), which is defined as a sinus heart rate >100 beats per minute at rest and a mean 24-h heart rate >90 beats per minute in the absence of secondary causes such as hyperthyroidism or anemia (Camara-Checa et al., 2023; Baruscotti et al., 2017; Bober et al., 2025).

Inappropriate sinus tachycardia has been reported to induce tachycardia-induced cardiomyopathy (TIC), a potentially reversible form of dilated cardiomyopathy resulting from sustained high heart rates (Winum et al., 2009; Sag et al., 2016; Romeo et al., 2011). Other arrhythmias more commonly associated with persistent tachycardia and the development of TIC include atrial flutter, atrial fibrillation, AV nodal re-entry tachycardia, ventricular extrasystoles, and ventricular tachycardia (Ellis and Josephson, 2013).

Given that HCN4 GOF mutations chronically elevate intrinsic heart rate, it might be hypothesized that such mutations could predispose to maladaptive cardiac remodeling including TIC. However, whether enhanced HCN4 activity *per se* is sufficient to trigger pathological remodeling or modify the cardiac response to hemodynamic stress remains unknown.

To address this question, we generated a knock-in mouse model carrying the HCN4(Y527 F) (hereafter HCN4F) GOF mutation in exon four of the Hcn4 gene which leads to increased channel activity and hence increased heart rates. Here, we used this model to test whether a chronic, HCN4-mediated increase in intrinsic heart rate is sufficient to induce structural or functional cardiac alterations, and whether it modifies the myocardial response to pressure overload. By combining *in vivo* and *ex vivo* analyses of cardiac electrophysiology, hemodynamics and morphology, we aimed to determine whether enhanced pacemaker activity predisposes to, or alters susceptibility towards, tachycardia-induced or pressure-induced remodeling. Surprisingly, our findings point towards an altered, and potentially protective, structural adaptation to hemodynamic stress, characterized by less ventricular dilatation and considerably preserved function.

## 2 Materials and methods

### 2.1 Animal model and ethical approval

All experiments were performed in accordance with institutional and national regulations and approved by the Regierung von Oberbayern (ROB, Munich, Germany). The study conformed to the Guide for the Care and Use of Laboratory Animals. The Hcn4(Y527 F) knock-in mouse line (HCN4F) was generated by targeted replacement of tyrosine 527 with phenylalanine in exon four of the Hcn4 gene. Mice were kept on a 12-h light/dark cycle with food and water *ad libitum*.

### 2.2 Heterologous expression in HEK293 Flp-In cells

WT and HCN4F channels were stably expressed in HEK293 Flp-In cells. Using a HEKA EPC10 amplifier and Patchmaster v2x90.2 (MCS GmbH), whole-cell patch clamp recordings were performed to determine steady-state activation curves, half-maximal activation voltage (V_0.5_), current densities, and the effect of intracellular cAMP (100 µM) on channel gating (Fenske et al., 2020). Data were analyzed using *OriginPro 2025 (OriginLab Corp.) and Fitmaster v2x90.2 (MCS GmbH)* software.

### 2.3 Telemetric ECG recordings

Continuous ECG recordings (60 h) were obtained in freely moving male WT and HCN4F mice (3 months old). Determined parameters included mean, minimum and maximum heart rate, and ECG intervals. Intraperitoneal administration of atropine (1 mg/kg BW) and propranolol (20 mg/kg BW) was used for autonomic blockade to determine intrinsic heart rate.

### 2.4 Langendorff-perfused isolated hearts

Hearts were excised and perfused retrogradely at constant pressure (80 mmHg). Spontaneous beating rate was determined from ECG recordings (Hennis et al., 2022b).

### 2.5 *In vivo* electrophysiological study (EPS)

*In vivo* EPS was performed under isoflurane anesthesia using a transvenous octapolar catheter introduced *via* the jugular vein into the right atrium and ventricle. Parameters determined included sinoatrial conduction time (SACT), sinus node recovery time (SNRT, cSNRT), atrioventricular nodal refractory period (AVNERP), atrial and ventricular effective refractory periods (AERP, VERP) (Hennis et al., 2022b).

### 2.6 Transverse aortic constriction

Pressure overload was induced in 10–12-week-old male mice by transverse aortic constriction (TAC) using a 27-G needle for calibration. Sham-operated controls were included. Hearts were analyzed 5 weeks post-surgery.

### 2.7 Pressure-volume loop analysis

Left ventricular pressure-volume loops were recorded in anaesthetized mice (13–15 months) using a Scisense 1.2 F Rodent pressure-volume catheter (Transonic Systems Inc.) connected to an ADV500 PV Loop System (Transonic Systems Inc.) and *LabChart 8 Pro* software (ADInstruments Ltd.). Data analysis in *LabChart* was carried out using the PV Loop analysis module. Parameters analyzed included end-diastolic and end-systolic volumes and pressures, dP/dt_max_, dP/dt_m_ᵢ_n_, time constant of diastolic relaxation τ, and ejection fraction.

### 2.8 Electrophysiology of isolated SAN cells

SAN cells were enzymatically digested from 8 to 12-week-old WT and HCN4F hearts. The SAN region was dissected and digested using a combination of collagenase B, elastase, and protease, followed by gentle mechanical dissociation. Isolated cells were kept in modified Kraft-Brühe solution for recovery before recording as described previously (Fenske et al., 2013; Fenske et al., 2016). Whole-cell and perforated-patch recordings were performed at 32 °C using a HEKA EPC10 amplifier and *Patchmaster v2x90.2* software (MCS GmbH). 100 μM cAMP was added to the intracellular solution as indicated. Data were analyzed using *OriginPro 2025 (OriginLab Corp.), Fitmaster v2x90.2 (MCS GmbH), and Clampfit* 10.5.2.6 (Molecular Devices, LLC.) software.

### 2.9 Electrophysiology of isolated ventricular myocytes

Ventricular myocytes were enzymatically isolated and their evoked action potentials recorded in current-clamp configuration as described previously (Fenske et al., 2011). Parameters determined included resting membrane potential and APD10/25/50/75/90. Repolarizing potassium currents (I_K_) were measured in voltage-clamp mode (Fenske et al., 2011). Whole-cell recordings were performed using a HEKA EPC10 amplifier and Patchmaster v2x90.2 software (MCS GmbH). Data were analyzed using *OriginPro 2025 (OriginLab Corp.), Fitmaster v2x90.2 (MCS GmbH), and Clampfit* 10.5.2.6 (Molecular Devices, LLC.) software.

### 2.10 Immunohistochemistry SAN cross sections

Cryosectioning and immunofluorescence staining of SAN tissue were performed as previously described (Fenske et al., 2020). SAN cross sections were stained with anti-HCN4 and anti-HCN1 antibodies, followed by FITC- and Cy3-conjugated secondary antibodies. Images were recorded using a Leica SP8 confocal microscope and analyzed using ImageJ (Schneider et al., 2012) to quantify fluorescence intensity (Fenske et al., 2013).

### 2.11 Whole-mount SAN preparation and immunofluorescence

Whole-mount SAN preparation and immunofluorescence stainings were performed as described previously (Fenske et al., 2013). Tissue was incubated with a guinea-pig polyclonal anti-HCN1 antibody (1:500; Alomone Labs) and rabbit polyclonal anti-HCN4 antibody (1:500; Alomone Labs). For the costaining, secondary antibodies Alexa555-conjugated anti-guinea pig (1:500; Invitrogen, Karlsruhe, Germany) and Alexa647-conjugated anti-rabbit (1:500; Invitrogen) were used. Immunofluorescent images were acquired using a Leica SP8 confocal microscope.

### 2.12 Histology

Hearts were fixed in 4% paraformaldehyde, embedded in paraffin, and sectioned coronally or transversally (Fenske et al., 2020). Sections were stained with hematoxylin and eosin or Sirius Red/Fast Green. Wall thickness (LV, RV, IVS), chamber cross-sectional areas, cardiomyocyte cross-section, and interstitial/perivascular fibrosis were quantified using light microscopy and ImageJ.

### 2.13 Quantitative PCR

Total RNA was isolated from left ventricular tissue. After reverse transcription, qPCR was performed for Nppa (ANP), Nppb (BNP), and Acta1. Expression levels were normalized to mALAS and shown relative to WT-Sham.

### 2.14 RNA sequencing (RNA-seq)

For RNA-seq, RNA isolated from LV tissue was used and sequenced on an Illumina platform; the same RNA samples were also employed for qPCR analyses. In total, 16 samples were analyzed, comprising four biological replicates per group (WT-Sham, HCN4F-Sham, WT-TAC, and HCN4F-TAC). Differential gene expression analysis (TAC *versus* Sham and WT *versus* HCN4F) was conducted using DESeq2. P-values and log2-fold changes were calculated using the Wald test. Genes with an adjusted p-value < 0.05 and an absolute log2-fold change > 1 were considered significantly differentially expressed.

### 2.15 Statistical analysis

Statistic analyses were performed using *Origin 2025* software (OriginLab Corporation, Northampton, MA, USA). Data are presented as mean ± SEM or box plots that show median (line), 25th–75th percentiles (box), minimum–maximum (whiskers), and mean (open circle). Statistical significance was determined by unpaired two-tailed t-test with Welch correction or two-way ANOVA with Fisher´s *post hoc* test, as appropriate. Left ventricular volume parameters derived from PV loop measurements were analyzed using a one-sided t-test, based on an *a priori* defined hypothesis. This hypothesis was founded on histological analyses demonstrating a consistent increase in left ventricular cavity size following TAC in both genotypes, with significantly smaller LV dimensions in HCN4F compared to WT after TAC surgery. The left ventricular catheter provides direct and very reliable pressure parameters using the pressure transducer, but less accurate absolute volume estimates due to inherent characteristics and limitations of conductance-based volume determination. One limitation might be that conductances (surrounding tissue) and geometrical assumptions could interfere with exact determination of ventricular volume leading to variability compared to histological analysis. We therefore relied on these independently validated histological data to define the expected direction of the effect. P < 0.05 was considered significant (P < 0.05 *, P < 0.01 **, P < 0.001 ***).

## 3 Results

### 3.1 Generation of the HCN4F mouse model

**FIGURE 1:**
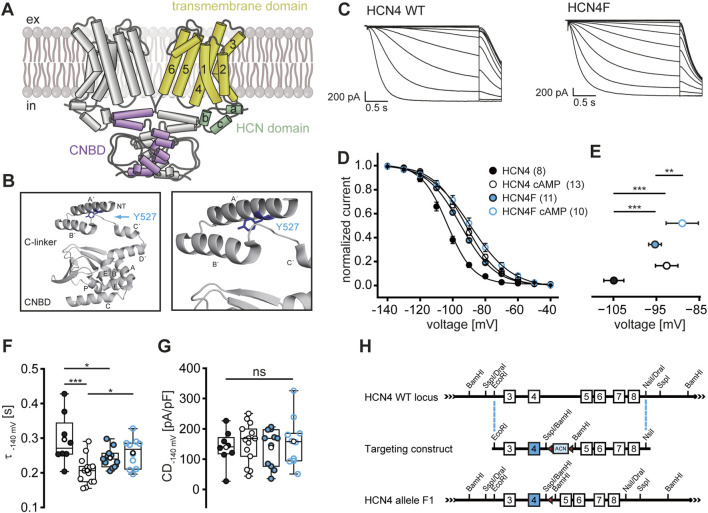
Characterization of the HCN4(Y527F) gain-of-function mutation and generation of the HCN4F mouse model. **(A)** Schematic depicting two subunits of the HCN4 channel tetramer, highlighting the transmembrane domains (yellow), C-Terminus comprising the cyclic nucleotide-binding domain (CNBD) and C-linker region (purple), and the N-terminal HCN domain (green). **(B)** Crystal structure of the human HCN4 C-terminal region (PDB: Q9Y3Q4) showing the arrangement of the A′ and B′ helices within the C-linker and the localization of the substitution of tyrosine 527 by phenylalanine. **(C)** Representative If current traces from HEK293 cells stably expressing WT (left) or HCN4F channels (right) under measurement conditions without cAMP. **(D)** Steady-state activation curves determined from measurements as shown in **(C)** for WT and HCN4F channels with (open circles) and without cAMP (filled circles). **(E)** Half-maximum activation voltage (V0.5) determined from steady-state activation curves. **(F)** Activation kinetics comparison between WT and HCN4F. **(G)** Mean current density of WT and HCN4F with and without cAMP. **(H)** Targeting strategy for generation of the HCN4F knock-in mouse line. The HCN4 WT locus comprises exons 3–8. Exon 4 depicted in blue carries the Hcn4Y527 F mutation. Data points in D and E represent the mean ± SEM; n numbers are given in parentheses. Box plots show median (line), 25th–75th percentiles (box), minimum–maximum (whiskers), and mean (open circle). P < 0.05 was considered significant (P < 0.05 *, P < 0.01 **, P < 0.001 ***).

To generate a GOF variant of the HCN4 channel, we rationally selected a mutation within the C-linker region based on the known structure of the human HCN4 C-terminus (PDB: Q9Y3Q4) (Figures 1A,B). Tyrosine 527, located in the A′ helix of the C-linker, was replaced by phenylalanine (HCN4Y527 F). This tyrosine residue is located in close proximity to glutamate 556 in the adjacent B′ helix (Figure 1B) and is predicted to participate in a hydrogen bond formation. Substitution of tyrosine by phenylalanine eliminates the hydroxyl group required for this interaction and is therefore expected to disrupt the hydrogen bond with glutamate 556. This potentially weakens inter-subunit interactions within the C-linker, which could facilitate conformational transitions within the C-linker region and thereby shift the voltage dependence of activation to more positive potentials.

To functionally assess the impact of this substitution, whole-cell patch-clamp recordings were performed in HEK293 cells stably expressing either wild-type (WT) or mutant HCN4 channels (Figure 1C). The steady-state activation curve of HCN4F was shifted by approximately 10 mV towards more positive potentials compared with WT (Figures 1D,E). Intracellular application of saturating cAMP concentration (100 µM) induced a significant rightward shift in the activation curve in both genotypes, demonstrating preserved cAMP-dependent regulation. Activation kinetics at −140 mV were significantly faster in HCN4F channels, and cAMP accelerated activation in WT but not in HCN4F (Figure 1F). Mean current density was similar among all groups (Figure 1G). Together, our findings confirm that the Y527 F substitution enhances channel availability at physiological membrane potentials.

To study the physiological consequences of this GOF mutation in vivo, a knock-in mouse line carrying the same substitution was generated by targeted modification of exon 4 within the Hcn4 gene (Figure 1H).

### 3.2 Validation of HCN4F channel expression and function in sinoatrial node tissue

**FIGURE 2:**
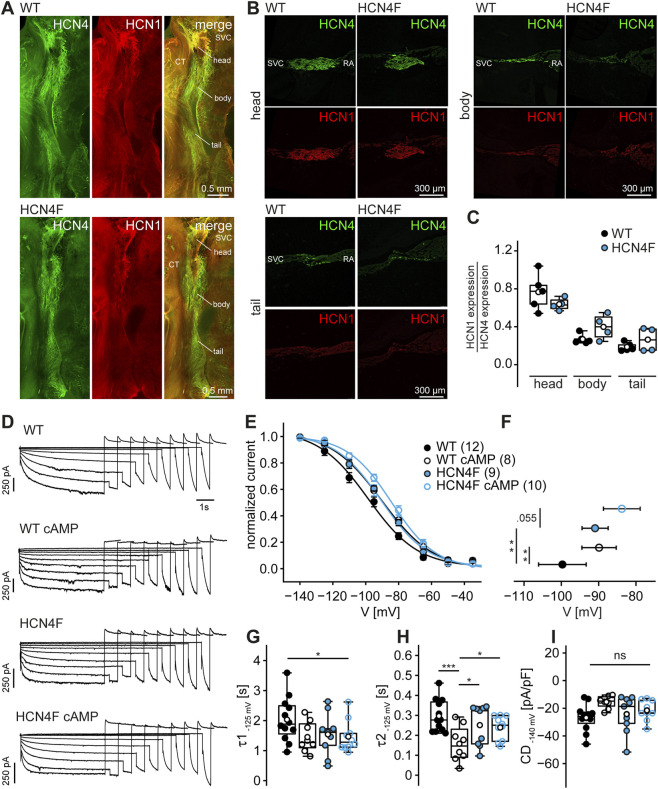
Validation of HCN4F channel expression and function in sinoatrial node tissue. **(A)** Representative whole-mount SAN immunofluorescence images of HCN4 (green) and HCN1 (red) staining from WT (top) and HCN4F (bottom). **(B)** Representative SAN section immunofluorescence images of HCN4 (green) and HCN1 (red) staining in head (top left), body (top, right) and tail region (bottom) of SAN tissue. **(C)** Quantification of intranodal HCN1/HCN4 area ratio in the head, body, and tail regions of the SAN. HCN channel distribution and expression levels are similar in WT and HCN4F SAN. **(D)** Representative families of If current traces recorded from isolated SAN cells of WT and HCN4F mice under baseline and cAMP (100 µM) conditions. **(E)** If steady-state activation curves determined from recordings as shown in **(C,F)** Half-maximal activation voltage (V0.5) comparison between WT and HCN4F under baseline and cAMP (100 µM) conditions. **(G,H)** Activation kinetics (τ1 and τ2) derived from double-exponential fits under baseline and cAMP conditions. **(I)** Mean If steady-state current density determined at −140 mV. Data are presented as mean ± SEM; n numbers are given in parentheses. SVC, superior caval vein; CT, crista terminalis; RA, right atrial appendage. Data points in E and F represent the mean ± SEM; n numbers are given in parentheses. Box plots show median (line), 25th–75th percentiles (box), minimum–maximum (whiskers), and mean (open circle). P < 0.05 was considered significant (P < 0.05 *, P < 0.01 **, P < 0.001 ***).

Protein distribution and expression levels of HCN4 and HCN1 channels, assessed by whole-mount and section immunofluorescence stainings in the head, body and tail regions of the SAN, were comparable between genotypes, indicating that the mutation does not alter the expression pattern or isoform composition (Figures 2A–C). Recordings of the hyperpolarization-activated current I_f_ from isolated SAN cells (Figure 2D) confirmed a significant depolarizing shift in the activation curve of ∼9 mV in HCN4F cells compared to WT (Figures 2E,F). Intracellular application of a saturating cAMP concentration induced an additional rightward shift of ∼7 mV in HCN4F cells, which did not reach statistical significance (p = 0.055), whereas WT cells exhibited a significant ∼10 mV shift. Although the cAMP-induced shift in HCN4F cells did not reach statistical significance, its direction and magnitude were consistent with preserved cAMP responsiveness, suggesting that the lack of significance is more likely due to limited statistical power than to a loss of cAMP sensitivity. The current density at −140 mV was similar among all four groups (WT, HCN4F, WT + cAMP, HCN4F + cAMP) (Figure 2I). Activation kinetics were determined by fitting a double-exponential function. The fast time constant (τ_2_) was similar across all groups, whereas the slow time constant (τ_1_) was faster in HCN4F cells compared to WT. Application of cAMP accelerated activation in WT but not in HCN4F cells (Figures 2G,H). Together, these data resemble the in vitro patch data in HEK293 cells and indicate that the Y527 F mutation increases channel availability at physiological membrane potentials (Figure 1). Hence, these results validate the HCN4F line as a bona fide HCN4 GOF mouse model to study the physiological and pathophysiological consequences of chronically enhanced pacemaker channel activity.

### 3.3 Higher intrinsic heart rate in HCN4F mice

**FIGURE 3:**
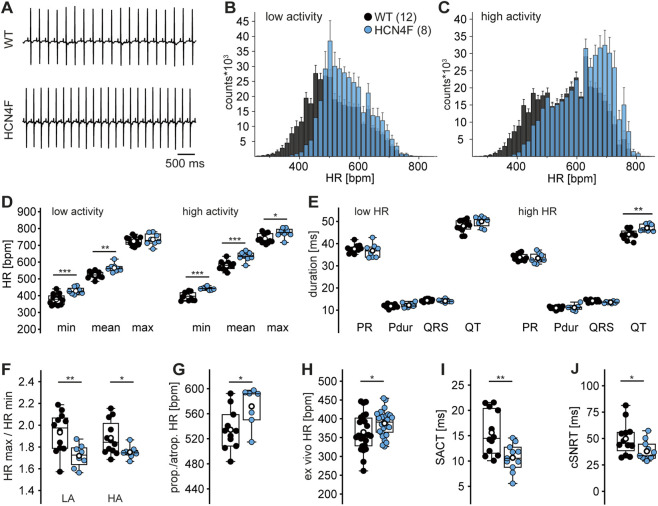
Higher intrinsic heart rate in HCN4F mice. **(A)** Representative telemetric ECG traces recorded in freely moving WT (top) and HCN4F mice (bottom). **(B,C)** Heart rate histograms of WT (black) and HCN4F (blue) showing distribution of HR across a 12-h light period (B; low activity) or dark period (C; high activity) recording period. HCN4F histograms are characterized by a reduced representation of low heart rate ranges and an increased proportion of higher heart rates, consistent with a mild IST-like phenotype. **(D)** Minimum, mean and maximum HR values in low and high activity phase. **(E)** ECG interval comparison at low HR (∼450 bpm) and high HR (∼650 bpm) showing mild QT prolongation in HCN4F at high HRs. **(F)** HR dynamics (HRmax/HRmin) during LA and HA phases. **(G)** HR after combined autonomic blockade with atropine and propranolol revealing higher intrinsic HR in HCN4F animals. **(H)** Spontaneous beating rate of isolated Langendorff-perfused hearts confirming higher intrinsic HR in HCN4F hearts. **(I,J)** Quantification of sinoatrial conduction time (SACT; **(I)** and corrected sinus node recovery time (cSNRT; **(J)** in WT and HCN4F mice from *in vivo* electrophysiological study. Box plots show median (line), 25th–75th percentiles (box), minimum–maximum (whiskers), and mean (open circle). P < 0.05 was considered significant (P < 0.05 *, P < 0.01 **, P < 0.001 ***); n numbers are given in parentheses.

Having established that the HCN4F mutation enhances pacemaker channel availability in SAN cells, we next examined whether this translates into changes of cardiac rhythm *in vivo*. Telemetric ECG recordings in freely moving mice demonstrated a stable sinus rhythm in WT and HCN4F animals (Figure 3A). HR histograms of HCN4F animals were truncated at lower heart rates during low-activity (LA) (Figure 3B) and high-activity (HA) phases, respectively (Figure 3C). Notably, HCN4F mice did not reach the lower heart rate range observed in WT animals, as reflected by the absence of histogram counts in the lowest frequency bins. Conversely, higher heart rate ranges were more frequently represented in HCN4F mice. Mean HR of HCN4F mice was approximately 10% higher than that of WT controls. Maximum HR values were similar (LA) or slightly increased (HA), whereas minimum HR values were higher in HCN4F mice, resulting in reduced HR dynamics (HRmax/HRmin) in both activity phases (Figures 3D,F).

ECG interval analysis showed no differences in PR interval, P-wave duration, or QRS duration between genotypes. At low HR (∼450 bpm), the QT interval was similar, but at high HR (∼650 bpm) it was slightly yet significantly prolonged in HCN4F mice (Figures 3E,F). To determine whether the mild QT interval elevation reflects a prolongation of ventricular repolarization at the cellular level, we assessed action potential parameters in isolated left-ventricular cardiomyocytes at 1 Hz stimulation. Resting membrane potential and APD10/25/50/75/90 were not significantly altered (Supplementary Figures 1A,B). In addition, densities of major repolarizing K^+^ currents were comparable between groups (Supplementary Figure 1C). Because ventricular AP parameters and major repolarizing K^+^ currents were unchanged, the mild QT prolongation observed in vivo is not explained by alteration at the cellular level. A likely explanation, which could be experimentally tested by optical mapping or electrophysiological experiments using multi electrode arrays in future analysis is that QT prolongation could arise from spatial heterogeneity in ventricular activation or repolarization at the tissue level.

Pharmacological blockade of the autonomic nervous system using atropine and propranolol revealed a higher intrinsic HR in HCN4F than in WT, confirming that the elevated HR originates from the SAN pacemaker rather than autonomic modulation (Figure 3G). This finding was corroborated in isolated Langendorff-perfused hearts, where ex vivo HCN4F hearts displayed a higher spontaneous beating rate (Figure 3H).

Furthermore, transvenous catheter-based in vivo electrophysiological study revealed reduced sinoatrial conduction time and reduced corrected sinus node recovery time following fast atrial overdrive-pacing (Figures 3I,J), indicating accelerated impulse formation and conduction within the SAN.

Together, these data indicate that the Y527 F mutation causes a mild but consistent sinus tachycardia due to an increase in intrinsic pacemaker activity independent of autonomic input.

### 3.4 Structural and histological analyses under basal conditions

**FIGURE 4:**
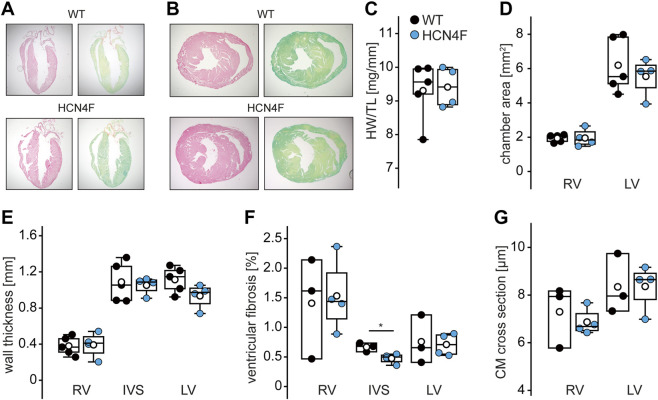
Structural and histological analysis of WT and HCN4F hearts under basal conditions. **(A,B)** Representative HE (left) and Sirius Red/Fast Green–stained (right) coronal **(A)** and transverse **(B)** heart sections of 6–7-month-old female mice indicating normal cardiac morphology in HCN4F hearts. **(C)** Heart weight/tibia length (HW/TL) ratios demonstrating unchanged heart weight in HCN4F mice. **(D)** Quantification of left and right ventricular chamber dimensions from coronal sections as shown in **(A,E)** Wall thickness of RV, IVS, and LV determined from coronal sections. **(F)** Quantification of fibrotic area from Sirius Red/Fast Green staining. **(G)** Cardiomyocyte cross-sectional area of RV and LV showing no genotype-dependent differences. Box plots show median (line), 25th–75th percentiles (box), minimum–maximum (whiskers), and mean (open circle). P < 0.05 was considered significant (P < 0.05 *, P < 0.01 **, P < 0.001 ***).

Having established that the HCN4F mutation increases intrinsic heart rate without inducing arrhythmias, we next examined whether chronic sinus tachycardia could cause tachycardia-induced cardiomyopathy or adverse structural remodeling of the heart. We performed detailed morphological and histological analyses. Hearts isolated from 6–7 months-old female mice showed no cardiac morphological abnormalities (Figures 4A,B), and heart weight to tibia length ratios were comparable between genotypes (Figure 4C).

Hematoxylin-eosin (HE) and Sirius Red/Fast Green staining were performed on coronal (Figure 4A) and transversal (Figure 4B) cross-sections to assess chamber dimensions, wall thickness, cardiomyocyte size, and collagen deposition. Both WT and HCN4F hearts exhibited normal left and right ventricular lumen dimensions (Figure 4D) and similar wall thicknesses of the right ventricle (RV), left ventricle (LV), and interventricular septum (IVS) (Figure 4E). No signs of interstitial or perivascular fibrosis of the LV or RV free wall, or IVS were detected in Sirius Red/Fast Green–stained sections (Figures 4A,B,F), and cardiomyocyte cross-sectional areas did not differ between genotypes (Figure 4G). Similar results were obtained in male mice aged 6–7 months as well as in 12-month-old animals of both sexes (data not shown).

Together, these findings demonstrate the absence of tachycardia-induced cardiomyopathy or other structural remodeling in HCN4F mice, indicating that the HCN4F GOF mutation and the resulting mild and chronic tachycardia do not exert adverse effects on cardiac structure up to 1 year of age.

### 3.5 Pressure overload reveals concentric rather than dilated remodeling in HCN4F hearts

**FIGURE 5:**
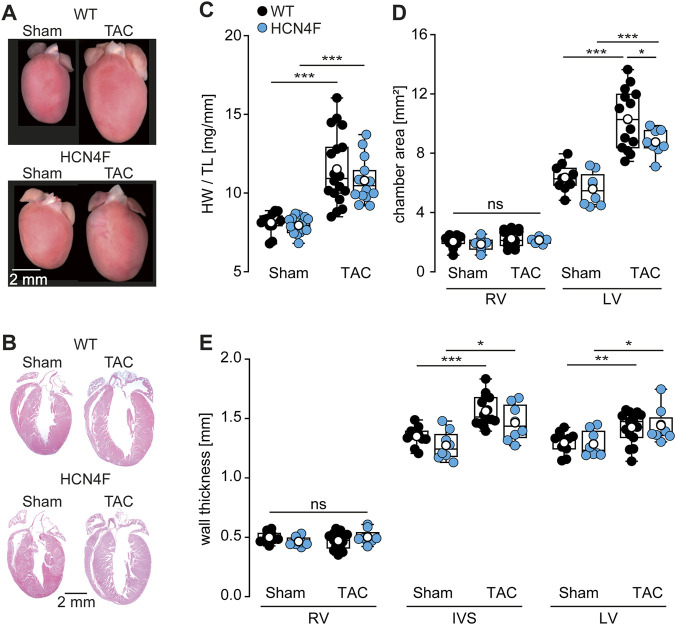
Morphological and histological characterization after pressure overload (TAC). **(A,B)** Representative gross morphology **(A)** and H&E-stained coronal heart sections **(B)** from WT (top) and HCN4F mice (bottom) after Sham (left) or TAC surgery (right), illustrating pressure overload–induced cardiac remodeling. **(C)** Heart weight normalized to tibia length significantly increased after TAC in both genotypes. **(D)** RV chamber area did not significantly increase after TAC, whereas LV chamber area was enlarged in both genotypes but remained significantly smaller in HCN4F mice, indicating reduced ventricular dilatation. **(E)** Interventricular septal (IVS), and left-ventricular (LV) wall thickness were similarly increased in WT and HCN4F hearts. RV wall thickness did not increase after TAC. Box plots show median (line), 25th–75th percentiles (box), minimum–maximum (whiskers), and mean (open circle). P < 0.05 was considered significant (P < 0.05 *, P < 0.01 **, P < 0.001 ***); n numbers HW/TL: WT Sham: 12; HCN4F Sham: 14; WT TAC: 18; HCN4F TAC: 14; n numbers chamber area and wall thickness: WT Sham: 12; HCN4F Sham: 8; WT TAC: 15; HCN4F TAC: 8.

Since structural and histological analyses revealed no evidence of tachycardia-induced cardiomyopathy in HCN4F mice, we next examined whether the chronically elevated heart rate might influence cardiac remodeling under stress conditions. To this end, WT and HCN4F mice were subjected to transverse aortic constriction (TAC) surgery, a model of pressure overload-induced hypertrophy (Figure 5). Four experimental groups were analyzed, comprising WT and HCN4F mice after either Sham or TAC surgery (Figures 5A,B). All animals survived the 5-week follow-up period after TAC surgery, and no deaths attributable to heart failure occurred during this time.

Heart weight/tibia length ratios increased significantly after TAC in both genotypes (Figure 5C) without genotype differences. LV wall and IVS thickness were significantly and equally increased in WT and HCN4F hearts following TAC surgery (Figure 5E). RV wall thickness did not increase upon TAC surgery in both genotypes. Morphometric analysis revealed that LV chamber area was significantly enlarged after TAC in both genotypes but remained significantly smaller in HCN4F mice (Figure 5D). Thus, HCN4F hearts displayed smaller LV lumen and less dilatation. RV chamber area did not increase upon TAC surgery in both genotypes.

Interstitial and perivascular fibrosis increased after TAC to a similar extent in both genotypes, indicating that HCN4F limits chamber dilatation without affecting hypertrophy or fibrosis (Supplementary Figure 2). In summary, the HCN4F mutation did not prevent TAC-induced hypertrophy or fibrosis, indicating that the phenotype reflects an altered remodeling pattern rather than a general prevention of pressure-overload remodeling.

**FIGURE 6:**
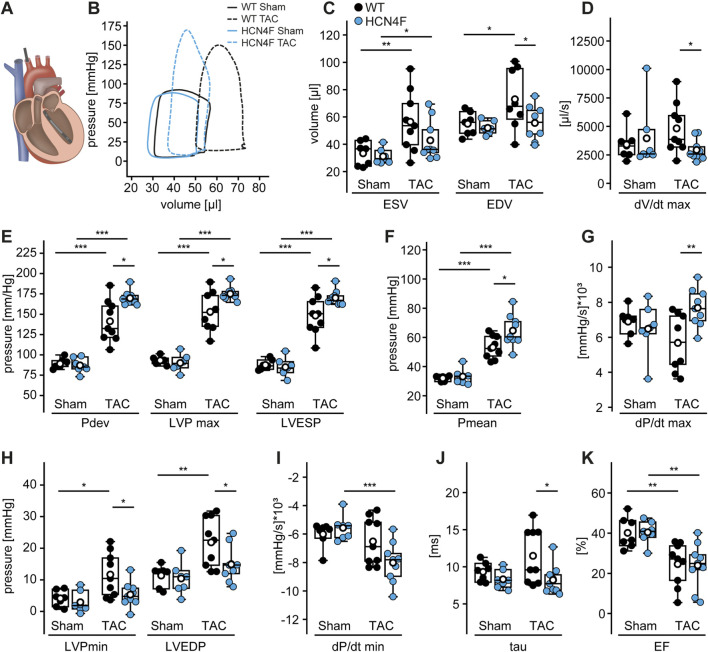
Hemodynamic analysis reveals preserved contractile performance and improved relaxation in HCN4F hearts after TAC. **(A)** Schematic depicting a pressure–volume (PV) loop catheter inserted into the left ventricle *via* the right common carotid artery. **(B)** Averaged PV loops determined from recordings of WT and HCN4F mice under sham or TAC conditions. **(C)** End-systolic (ESV) and end-diastolic volumes (EDV) showing reduced EDV in HCN4F-TAC hearts compared to WT-TAC hearts. **(D)** Maximal rate of volume change (dV/dtmax) was lower in HCN4F-TAC hearts. **(E,F)** Systolic pressure parameters (Pdev, LVPmax, LVESPP, Pmean) increased after TAC in both genotypes, with higher values in HCN4F-TAC mice. **(G)** Contractility (dP/dt_max) was enhanced in HCN4F-TAC compared with WT-TAC hearts. **(H,I)** Diastolic pressures (LVPmin, LVEDP) in HCN4F-TAC hearts. **(I)** More negative dP/dtmin in HCN4F-TAC hearts. **(J)** The relaxation time constant (tau) was significantly shorter in HCN4F-TAC hearts. **(K)** Ejection fraction (EF) decreased after TAC in both genotypes but was better preserved in HCN4F mice. Box plots show median (line), 25th–75th percentiles (box), minimum–maximum (whiskers), and mean (open circle). P < 0.05 was considered significant (P < 0.05 *, P < 0.01 **, P < 0.001 ***); n numbers: WT Sham: 7; HCN4F Sham: 7; WT TAC: 9; HCN4F TAC: 9.

To determine whether the structural differences observed after TAC are accompanied by altered cardiac function, we performed in vivo pressure–volume (PV) loop analysis in anaesthetized mice (Figures 6A,B).

Under sham conditions, hemodynamic parameters were comparable between genotypes (Figures 6C–K). Following TAC, both WT and HCN4F mice exhibited the expected increase in left ventricular pressure parameters, confirming successful induction of pressure overload.

In WT mice, TAC led to a significant increase in end-systolic volume (ESV) and a trend towards increased end-diastolic volume (EDV), indicating ventricular dilatation (Figure 6C). In contrast, neither ESV nor EDV increased significantly in HCN4F mice after TAC. These in vivo findings are in agreement with the morphometric analysis showing smaller LV chamber areas in HCN4F mice after TAC (Figure 5D). The maximal rate of volume change (dV/dtmax) was significantly lower in HCN4F-TAC compared with WT-TAC hearts, consistent with the smaller increases in ventricular volumes observed in HCN4F mice after pressure overload (Figure 6D).

Analysis of systolic pressure parameters revealed a pronounced pressure generation in HCN4F hearts after TAC. While TAC significantly increased developed pressure (Pdev), maximal left ventricular pressure (LVPmax), end-systolic pressure (LVESP), and mean pressure (Pmean) in both genotypes compared with their respective sham controls, all of these parameters were significantly higher in HCN4F-TAC than in WT-TAC mice (Figures 6E,F). Consistent with these findings, contractility assessed by dP/dtmax was significantly higher in HCN4F-TAC hearts compared with WT-TAC hearts (Figure 6G).

Differences between genotypes were also observed in diastolic pressure parameters. TAC elevated diastolic pressures in both genotypes, but LVPmin and LVEDP remained significantly lower in HCN4F-TAC than in WT-TAC hearts (Figure 6H). In HCN4F mice, TAC induced a significant decrease in dP/dtmin (Figure 6I), whereas no significant change was detected in WT mice. Furthermore, the relaxation time constant τ was significantly shorter in HCN4F-TAC compared with WT-TAC hearts, indicating faster relaxation kinetics (Figure 6I).

Finally, ejection fraction (EF) was significantly reduced by TAC in both genotypes (Figure 6K).

Together, these hemodynamic data indicate that TAC induces ventricular dilatation predominantly in WT hearts, whereas HCN4F hearts maintain smaller ventricular volumes while generating higher systolic pressures and enhanced contractility. These functional findings are consistent with the histological observations of comparable hypertrophy but reduced chamber enlargement in HCN4F mice, supporting the concept of a more concentric pattern of remodeling under pressure overload.

### 3.6 Cardiac hypertrophy markers and transcriptome analysis

To obtain an overview of transcriptional remodeling, RNA-Seq was performed on left ventricular samples from WT and HCN4F mice subjected to Sham or TAC surgery.

RNA-Seq and differential expression analyses revealed a broad transcriptional reprogramming in both genotypes following TAC, including activation of pathways related to hypertrophy, extracellular matrix remodeling, inflammation, and stress response (Supplementary Table 1). Analysis of classical hypertrophic markers revealed that the expression of genes of the fetal gene program such as atrial natriuretic peptide (Nppa) and brain natriuretic peptide (Nppb) were upregulated in both genotypes, confirming the effective induction of pressure-overload hypertrophy, but they did not differ between HCN4F-TAC and WT-TAC groups. In contrast, a significant increase in skeletal muscle α-actin (Acta1; ∼2.8-fold) in HCN4F-TAC hearts was detected. To validate these RNA-Seq results, mRNA levels of Nppa, Nppb, and Acta1 were quantified by qPCR. Consistent with the transcriptomic data, ANP and BNP expression increased to a similar extent in WT and HCN4F hearts, whereas Acta1 expression was approximately twofold higher in HCN4F-TAC ventricles. No genotype differences were observed under Sham conditions.

While the overall response to TAC surgery was similar between WT and HCN4F mice, several genes exhibited genotype-specific regulation following TAC surgery, suggesting differences in the adaptive remodeling process. In total, 64 genes were significantly differentially expressed between the two TAC groups, with the majority being upregulated in HCN4F-TAC hearts (47 upregulated vs. 17 downregulated genes; log2FC > 1; padj < 0.05).

Among the upregulated genes in HCN4F-TAC versus WT-TAC hearts, several are known to be associated with mechanoadaptive signaling pathways and extracellular matrix (ECM) remodeling, including Vgll2 (log2FC: 2.8), Timp1 (log2FC: 2.9), Ankrd1 (log2FC: 2.2), Crlf1 (log2FC: 2.1), Has1 (log2FC: 2.2), Col12a1 (log2FC: 1.5), and Col8a1/2 (log2FC: 1.2). In parallel, increased Nrg1 (log2FC: 1.3) and Gdf15 (log2FC: 1.9) expression may point to cardioprotective signaling that favors compensated remodeling. In addition, expression of the pro-inflammatory chemokines Ccl12 (log2FC: 3.7), Ccl2 (log2FC: 2.9), and Ccl7 (log2FC: 2.5) was significantly increased, suggesting activation of inflammatory signaling pathways and increased recruitment of immune cells in HCN4F-TAC hearts.

Together, these findings confirm that the HCN4F mutation does not prevent activation of the hypertrophic program but slightly alters its magnitude and composition, potentially contributing to a more adaptive transcriptional phenotype that may underlie the improved ventricular performance observed in HCN4F mice after pressure overload.

## 4 Discussion

### 4.1 Basal phenotype of HCN4F animals

HCN4F mice displayed a mild sinus tachycardia with a ∼10% increase in heart rate compared to WT controls, consistent with the gain-of-function nature of the HCN4 mutation. No evidence of tachycardia-induced cardiomyopathy was observed, likely due to the only mild elevation in heart rate. Heart size, wall thickness, fibrosis, and hypertrophy marker expression were comparable between genotypes, and LV function assessed by hemodynamic measurements was normal. The absence of TIC likely reflects the modest heart rate elevation, which is considerably smaller than in pacing models where the rate is nearly doubled (Ellis and Josephson, 2013; Shinbane et al., 1997), and the limited susceptibility of mice with high basal heart rates to develop frequency-induced cardiomyopathy (Milani-Nejad and Janssen, 2014).

### 4.2 Response to pressure overload

To assess the impact of sinus tachycardia and HCN4 channel activity on the adaptation to pressure overload, mice underwent transverse aortic constriction. As expected, TAC induced hypertrophy in both genotypes, but the degree and pattern of remodeling differed markedly. WT hearts showed pronounced LV dilation and eccentric hypertrophy, whereas HCN4F hearts exhibited a more compact geometry with smaller ventricular cavities, consistent with concentric hypertrophy. Fibrosis increased to a similar extent in both groups, indicating that differences in outcome were not related to differences in fibrosis. The lack of LV dilation in HCN4F mice despite comparable heart weights suggests more efficient adaptation to pressure overload.

### 4.3 Distinct hemodynamic responses to pressure overload

PV loop analysis confirmed systolic dysfunction and reduced ejection fraction and cardiac output after TAC in both genotypes. However, HCN4F mice maintained higher systolic pressures and exhibited increased contractility (dP/dtmax) and faster relaxation (dP/dtmin, tau) compared to WT, despite similar overall stroke work. The smaller LV cavity (smaller ventricular radius r) in HCN4F mice would increase systolic pressure P according to the Laplace relationship ( $P=\frac{\sigma }{r}\cdot 2d$ with radius r, wall stress σ, pressure P, and wall thickness d), potentially facilitating pressure generation under pressure overload conditions with unchanged or even reduced wall stress σ.

Together, these hemodynamic data demonstrate that while both WT and HCN4F mice develop pressure-induced hypertrophy, the pattern of remodeling differs. WT hearts undergo dilated remodeling with impaired contractility, whereas HCN4F hearts maintain higher systolic pressures, faster relaxation, and smaller chamber volumes—indicative of concentric rather than dilated remodeling. This suggests that chronic higher HCN4 activity supports an adaptive and efficient response to pressure overload, limiting maladaptive ventricular dilatation.

### 4.4 Distinct transcriptional responses to pressure overload

Transcriptomic analysis revealed a distinct gene expression signature in HCN4F-TAC hearts compared with WT-TAC hearts, characterized by increased expression of genes associated with hypertrophic adaptation, ECM remodeling, and stress-responsive signaling (a detailed gene-by-gene analysis is provided in the Supplementary Discussion). Upregulation of Acta1 and Vgll2 in HCN4F-TAC hearts could contribute to the increased contractility and elevated pressure values observed in PV loop measurements. In parallel, increased expression of Nrg1, Gdf15, and Crlf1 points to activation of pro-survival and stress-adaptive signaling pathways. Several genes involved in ECM organization, including Col12a1, Timp1, and Has1, were also elevated, indicating distinct matrix remodeling. In addition, transcripts related to inflammatory signaling (Ccl2, Ccl7, and Ccl12) were increased, consistent with activation of remodeling-associated stress responses.

Together, these findings indicate a distinct transcriptional response to pressure overload in HCN4F hearts that may contribute to preserved cardiac function and altered remodeling compared with WT-TAC. The transcriptomic findings describe how identified candidate pathways correlate with the altered remodeling phenotype, rather than establishing direct causal links between elevated intrinsic pacemaking and the observed gene expression program. The distinct responses could arise as direct consequence of increased intrinsic pacing rate, altered wall stress, altered HCN4-dependent signaling, or other secondary adaptations. These possibilities were not further evaluated in the present study.

### 4.5 Clinical implications

The different remodeling patterns of WT and HCN4F hearts under pressure overload suggest that enhanced pacemaker activity and mild sinus tachycardia improve adaptive remodeling. By preventing LV dilation and sustaining systolic performance, HCN4F mice appear protected from the transition to decompensated HF.

At first glance, this observation seems counterintuitive, as elevated heart rate is generally associated with adverse outcomes in HF, and lowing heart rate, for example, through β-blockers or ivabradine is a therapeutic measure. In HF patients, tachycardia commonly reflects chronic activation of the sympathetic nervous system and increased neurohumoral activity, both hallmarks of progressive HF that promote maladaptive remodeling and arrhythmogenesis. In contrast, the elevated heart rate observed in HCN4F mice results from enhanced intrinsic pacemaker activity rather than sustained neurohumoral activity. A similar distinction may apply to pacing-induced increases in heart rate in HF patients. For example, recent clinical studies in patients with HF with preserved ejection fraction (HFpEF) have suggested that a moderate increase in pacemaker rate can improve functional capacity and clinical outcome, presumably by improving ventricular filling dynamics and cardiac output during exertion (Wahlberg et al., 2019; Infeld et al., 2023; Klein et al., 2016). Our study indicates that in pressure-overload induced HF with reduced ejection fraction (HFrEF) a moderate increase in intrinsic pacemaker activity could also exert beneficial effects in HFrEF conditions by limiting maladaptive chamber dilatation. On the other hand, it has long been recognized that a significant increase in heart rate is associated with a marked worsening of clinical symptoms, reduced life expectancy, and poorer outcomes in patients with HFrEF. Therefore, future studies may help identify a therapeutic window in which mildly elevated heart rates confer benefit in specific subgroups of patients with HFrEF.

Together with other studies which have reported neutral or even detrimental effects when pacing rates were increased in HFpEF patients (Oraii et al., 2024) the observations described emphasize that the therapeutic window between beneficial and maladaptive chronotropy may be narrow and that in patients, different intrinsic heart rates could underlie interindividual variability in HF progression.

## Data Availability

The data presented in the study are deposited in the GEO repository, accession number GSE329403.
