# Supplementary material for: HCN4 gain-of-function mutation increases intrinsic heart rate and limits maladaptive remodeling under pressure overload
Source: Front Pharmacol. 2026 Jun 10;17:1840832. doi: 10.3389/fphar.2026.1840832 (PMC13291042; doi:10.3389/fphar.2026.1840832)
Supplement: Supplementary file 1 [file DataSheet1.docx]

Supplementary Material

# Supplementary Figures


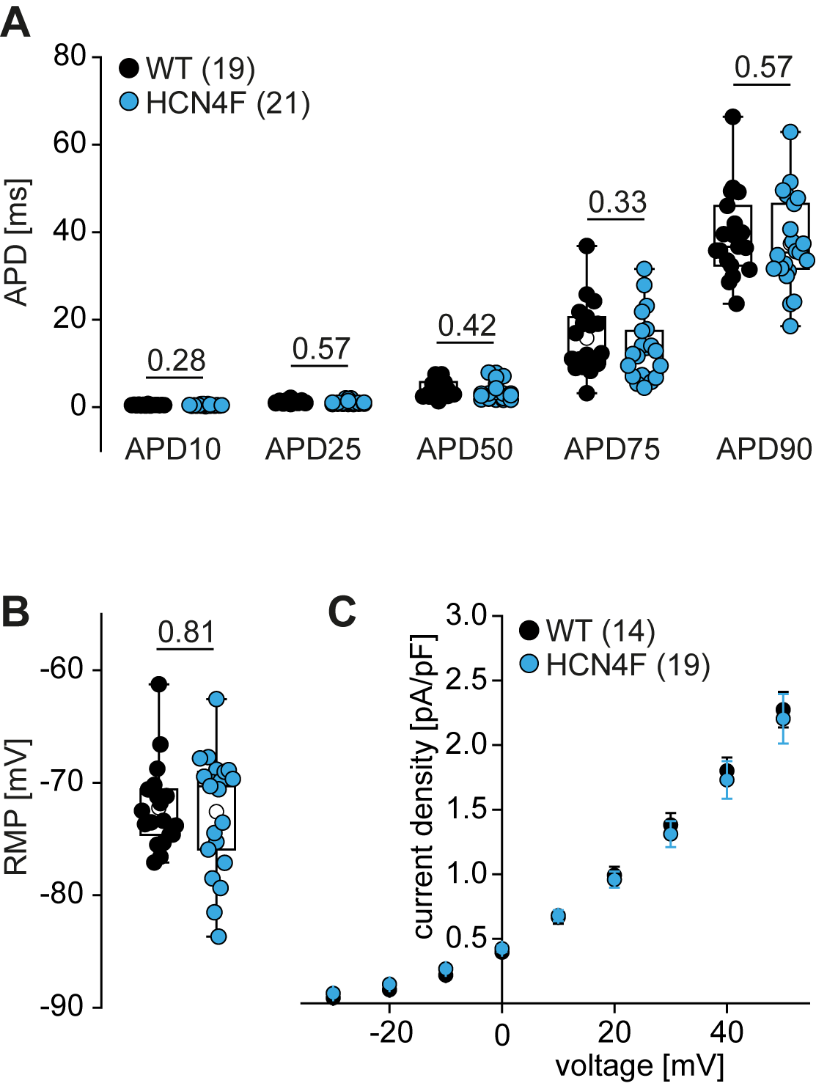


**Supplementary Figure 1.** Electrophysiological parameters in ventricular cardiomyocytes (*A*) APD10/25/50/75/90 determined in left ventricular cardiomyocytes showing no significant differences between WT and HCN4F cells. (*B*) Resting membrane potential determined in WT and HCN4F left ventricular cardiomyocytes. (*C*) Current density of major repolarizing K⁺ currents in WT and HCN4F cardiomyocytes showing no significant differences between WT and HCN4F cells. For *A, B*, box plots display median (line), 25th–75th percentiles (box), minimum–maximum (whiskers), and mean (open circle). For *C*, data are presented as mean ± SEM. P < 0.05 was considered significant (P < 0.05 *, P < 0.01 **, P < 0.001 ***); n numbers are given in parentheses.


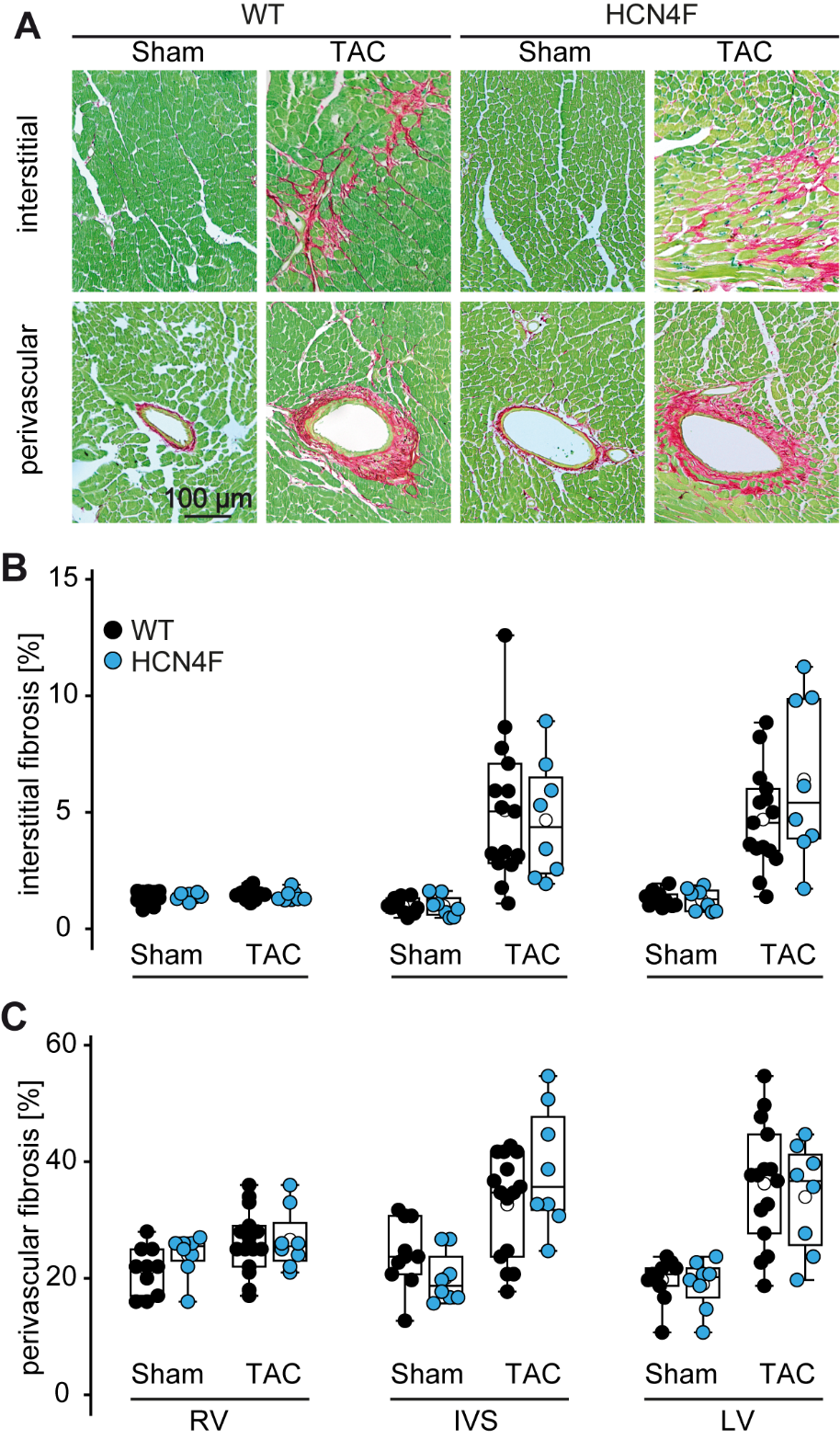


Supplementary Figure 2. Histological characterization after pressure overload (TAC) *(A)* Sirius Red/Fast Green staining of left-ventricular sections from WT and HCN4F mice after Sham or TAC surgery illustrating interstitial (upper panels) and perivascular (lower panels) fibrosis. *(B)* Quantification of interstitial fibrosis (%) in RV, IVS, and LVV from Sirius Red/Fast Green–stained sections of WT and HCN4F hearts after Sham and TAC surgery. *(C)* Quantification of perivascular fibrosis (%) in LV, IVS, and RV from Sirius Red/Fast Green–stained sections of WT and HCN4F hearts after Sham and TAC surgery. Box plots display median (line), 25th–75th percentiles (box), minimum–maximum (whiskers), and mean (open circle). P < 0.05 was considered significant (P < 0.05 *, P < 0.01 **, P < 0.001 ***). WT Sham: 11; HCN4F Sham: 8; WT TAC: 15; HCN4F TAC: 9.

# Supplementary discussion

Increased *Acta1* expression in HCN4F-TAC hearts may reflect a compensatory structural adaptation of cardiomyocytes to elevated mechanical load. *Acta1* encodes α-skeletal actin, a sarcomeric thin-filament isoform that is re-induced in the heart as part of the fetal/hypertrophic gene program induced by pressure overload. In cardiomyocytes, sustained mechanical stress promotes actin-dependent sarcomere assembly and parallel addition of myofilaments to preserve force generation under increased systolic load. Moreover, higher cardiac α-skeletal actin content has been associated with increased myocardial contractility in mice (Hewett et al., 1994), indicating that *Acta1* induction may support pressure generation in pressure-overload myocardium. The higher *Acta1* expression observed in HCN4F-TAC hearts could therefore contribute to the increased contractility and elevated pressure values observed in PV loop measurements.

Vestigial-like family member 2 (Vgll2) encodes a transcriptional cofactor that regulates muscle gene programs through interaction with TEAD transcription factors and has been implicated in functional adaptations to chronic mechanical load. Experimental studies in muscle tissue indicate that VGLL2 promotes oxidative metabolism and mitochondrial function (Yeshwant et al., 2019), processes that support contractile performance and cellular stress tolerance. Thus, the increased Vgll2 expression observed in HCN4F-TAC hearts may reflect activation of adaptive muscle gene programs contributing to improved myocardial function.

Concomitant increases in Neuregulin 1 (Nrg1) and growth differentiation factor 15 (Gdf15) expression in HCN4F-TAC hearts further points to activation of pro-survival signaling. NRG1 has been shown to enhance systolic function during pressure overload while promoting adaptive concentric remodeling without increasing cardiomyocyte hypertrophy. In TAC models, NRG1 prevents early cardiac decompensation (Xu et al., 2026). Consistent with these findings, clinical studies in patients with chronic heart failure (HF) demonstrated that recombinant NRG1 therapy improves left-ventricular function and reduces ventricular volumes, indicating reversal of pathological remodeling (Gao et al., 2010, Jabbour et al., 2011). Together, these observations suggest that increased *Nrg1* expression may contribute to the more adaptive remodeling response observed in HCN4F-TAC hearts. Overexpression of *Gdf15* protects cardiomyocytes from agonist-induced hypertrophy and attenuates pressure overload–induced cardiac hypertrophy in transgenic mice, whereas *Gdf15*-deficient mice develop exaggerated hypertrophy under similar conditions (Xu et al., 2006, Emmerson et al., 2018). The increased *Gdf15* expression observed in HCN4F-TAC hearts may therefore, like *Nrg1*, contribute to the more adaptive hypertrophic response compared with WT-TAC hearts.

Additionally, upregulation of Col12a1 in HCN4F-TAC hearts compared with WT-TAC hearts indicates adaptive ECM reorganization that may enhance mechanical stability without promoting fibrosis (Izu and Birk, 2023). Collagen XII is a non-fibrillar matrix-bridging protein that provides in regenerative models (Marro et al., 2016), a transient scaffold supporting tissue repair and cell migration, suggesting that increased Col12a1 expression during pressure overload may reflect adaptive ECM remodeling that stabilizes myocardial structure and facilitates compensatory tissue reorganization rather than permanent fibrotic scarring.

*Timp1* upregulation has been linked to both limitation of post-infarct adverse remodeling (Creemers et al., 2003, Glass and Singla, 2012, Uchinaka et al., 2014) and promotion of fibrosis under chronic pressure overload (Heymans et al., 2005), its functional contribution in our model remains difficult to define and may depend on the specific remodeling context.

Ankrd1 expression is rapidly induced in response to mechanical stress and pressure overload and is frequently elevated in cardiac hypertrophy (Aihara et al., 2000). Experimental studies suggest that ANKRD1 may act as a stress-responsive regulator of hypertrophic signaling. Transgenic overexpression attenuated pressure overload–induced hypertrophy through inhibition of ERK and TGF-β/Smad pathways (Song et al., 2012), whereas other studies reported exacerbated pathological remodeling via activation of the calcineurin–NFAT pathway (Chen et al., 2014). Thus, although the functional role of ANKRD1 in cardiac hypertrophy remains debated, increased Ankrd1 expression in HCN4F-TAC hearts may contribute to adaptive remodeling.

Increased expression of Crlf1 in HCN4F-TAC hearts may indicate activation of cytokine-mediated stress response pathways. CRLF1 forms a cytokine complex that activates gp130-dependent JAK/STAT signaling (Crisponi et al., 2022), and activation of the gp130/STAT3 pathway has been shown to promote cardiomyocyte survival and protect the heart from stress-induced injury (Hilfiker-Kleiner et al., 2005).

Elevated expression of the cytoprotective enzyme hyaluronan synthase 1 (HAS1) may support cardiomyocyte survival under pressure overload. HAS1 is one of three isoenzymes responsible for hyaluronan synthesis. In murine models of myocardial infarction increased hyaluronan production contributes to post-infarct healing by supporting macrophage survival and promoting the activation of reparative myofibroblasts (Siiskonen et al., 2015, Petz et al., 2019). Through similar mechanisms, enhanced HAS1 activity may stabilize ECM, modulate inflammatory signaling, and facilitate adaptive remodeling in HCN4F-TAC hearts.

In addition to genes associated with extracellular matrix remodeling and hypertrophic signaling, several transcripts related to inflammatory and wound-healing responses were increased in HCN4F-TAC hearts, including Ccl12, Ccl2, and Ccl7. Chemokines and inflammatory mediators are known to be induced during pressure overload (Patel et al., 2018) and contribute to cardiac remodeling by regulating immune cell recruitment, fibroblast activation, and ECM composition. The increased expression of these genes in HCN4F-TAC hearts may therefore reflect activation of stress-responsive signaling pathways associated with tissue remodeling following pressure overload.

Together, the distinct gene expression pattern in HCN4F-TAC hearts may suggest an enhanced adaptive and cardioprotective response in HCN4F-TAC hearts, that could contribute to the preserved function under chronic pressure overload. However, the mechanisms by which the HCN4F mutation drives these transcriptional changes and remodeling processes remain an important subject for future studies.

# Supplementary References

Aihara, Y., Kurabayashi, M., Saito, Y., Ohyama, Y., Tanaka, T., Takeda, S., et al. 2000. Cardiac ankyrin repeat protein is a novel marker of cardiac hypertrophy: role of M-CAT element within the promoter. Hypertension, 36**,** 48-53.10.1161/01.hyp.36.1.48

Chen, C., Shen, L., Cao, S., Li, X., Xuan, W., Zhang, J., et al. 2014. Cytosolic CARP promotes angiotensin II- or pressure overload-induced cardiomyocyte hypertrophy through calcineurin accumulation. PLoS One, 9**,** e104040.10.1371/journal.pone.0104040

Creemers, E. E., Davis, J. N., Parkhurst, A. M., Leenders, P., Dowdy, K. B., Hapke, E., et al. 2003. Deficiency of TIMP-1 exacerbates LV remodeling after myocardial infarction in mice. Am J Physiol Heart Circ Physiol, 284**,** H364-71.10.1152/ajpheart.00511.2002

Crisponi, L., Buers, I. & Rutsch, F. 2022. CRLF1 and CLCF1 in Development, Health and Disease. Int J Mol Sci, 23.10.3390/ijms23020992

Emmerson, P. J., Duffin, K. L., Chintharlapalli, S. & Wu, X. 2018. GDF15 and Growth Control. Front Physiol, 9**,** 1712.10.3389/fphys.2018.01712

Gao, R., Zhang, J., Cheng, L., Wu, X., Dong, W., Yang, X., et al. 2010. A Phase II, randomized, double-blind, multicenter, based on standard therapy, placebo-controlled study of the efficacy and safety of recombinant human neuregulin-1 in patients with chronic heart failure. J Am Coll Cardiol, 55**,** 1907-14.10.1016/j.jacc.2009.12.044

Glass, C. & Singla, D. K. 2012. Overexpression of TIMP-1 in embryonic stem cells attenuates adverse cardiac remodeling following myocardial infarction. Cell Transplant, 21**,** 1931-44.10.3727/096368911X627561

Hewett, T. E., Grupp, I. L., Grupp, G. & Robbins, J. 1994. Alpha-skeletal actin is associated with increased contractility in the mouse heart. Circ Res, 74**,** 740-6.10.1161/01.res.74.4.740

Heymans, S., Schroen, B., Vermeersch, P., Milting, H., Gao, F., Kassner, A., et al. 2005. Increased cardiac expression of tissue inhibitor of metalloproteinase-1 and tissue inhibitor of metalloproteinase-2 is related to cardiac fibrosis and dysfunction in the chronic pressure-overloaded human heart. Circulation, 112**,** 1136-44.10.1161/CIRCULATIONAHA.104.516963

Hilfiker-Kleiner, D., Hilfiker, A. & Drexler, H. 2005. Many good reasons to have STAT3 in the heart. Pharmacol Ther, 107**,** 131-7.10.1016/j.pharmthera.2005.02.003

Izu, Y. & Birk, D. E. 2023. Collagen XII mediated cellular and extracellular mechanisms in development, regeneration, and disease. Front Cell Dev Biol, 11**,** 1129000.10.3389/fcell.2023.1129000

Jabbour, A., Hayward, C. S., Keogh, A. M., Kotlyar, E., McCrohon, J. A., England, J. F., et al. 2011. Parenteral administration of recombinant human neuregulin-1 to patients with stable chronic heart failure produces favourable acute and chronic haemodynamic responses. Eur J Heart Fail, 13**,** 83-92.10.1093/eurjhf/hfq152

Marro, J., Pfefferli, C., de Preux Charles, A. S., Bise, T. & Jazwinska, A. 2016. Collagen XII Contributes to Epicardial and Connective Tissues in the Zebrafish Heart during Ontogenesis and Regeneration. PLoS One, 11**,** e0165497.10.1371/journal.pone.0165497

Patel, B., Bansal, S. S., Ismahil, M. A., Hamid, T., Rokosh, G., Mack, M., et al. 2018. CCR2(+) Monocyte-Derived Infiltrating Macrophages Are Required for Adverse Cardiac Remodeling During Pressure Overload. JACC Basic Transl Sci, 3**,** 230-244.10.1016/j.jacbts.2017.12.006

Petz, A., Grandoch, M., Gorski, D. J., Abrams, M., Piroth, M., Schneckmann, R., et al. 2019. Cardiac Hyaluronan Synthesis Is Critically Involved in the Cardiac Macrophage Response and Promotes Healing After Ischemia Reperfusion Injury. Circ Res, 124**,** 1433-1447.10.1161/CIRCRESAHA.118.313285

Siiskonen, H., Oikari, S., Pasonen-Seppanen, S. & Rilla, K. 2015. Hyaluronan synthase 1: a mysterious enzyme with unexpected functions. Front Immunol, 6**,** 43.10.3389/fimmu.2015.00043

Song, Y., Xu, J., Li, Y., Jia, C., Ma, X., Zhang, L., et al. 2012. Cardiac ankyrin repeat protein attenuates cardiac hypertrophy by inhibition of ERK1/2 and TGF-beta signaling pathways. PLoS One, 7**,** e50436.10.1371/journal.pone.0050436

Uchinaka, A., Kawaguchi, N., Mori, S., Hamada, Y., Miyagawa, S., Saito, A., et al. 2014. Tissue inhibitor of metalloproteinase-1 and -3 improves cardiac function in an ischemic cardiomyopathy model rat. Tissue Eng Part A, 20**,** 3073-84.10.1089/ten.TEA.2013.0763

Xu, J., Kimball, T. R., Lorenz, J. N., Brown, D. A., Bauskin, A. R., Klevitsky, R., et al. 2006. GDF15/MIC-1 functions as a protective and antihypertrophic factor released from the myocardium in association with SMAD protein activation. Circ Res, 98**,** 342-50.10.1161/01.RES.0000202804.84885.d0

Xu, L., Aghagolzadeh, P., Morandi, C., Wagner, J., Lepine, L. M., Segers, V. F. M., et al. 2026. Neuregulin-1beta augments adaptive concentric remodeling and systolic function without exacerbating hypertrophy during pressure overload. Am J Physiol Heart Circ Physiol, 330**,** H818-H837.10.1152/ajpheart.00371.2025

Yeshwant, S. C., Zile, M. R., Lewis, M. R., Lewinter, M. & Meyer, M. 2019. Safety and Feasibility of a Nocturnal Heart Rate Elevation-Exploration of a Novel Treatment Concept. J Card Fail, 25**,** 67-71.10.1016/j.cardfail.2018.06.009

# Supplementary Table

**RNA-Sequencing Analysis**

**Differentially expressed genes in the left ventricle (WT-Sham vs. HCN4F-Sham)**

| Gen Symbol | Gen ID | log2FoldChange | p-value | Padj |
| --- | --- | --- | --- | --- |
| Slc15a2 | ENSMUSG00000022899 | 3,83176 | 7,05E-09 | 3,26E-06 |
| Egr1 | ENSMUSG00000038418 | 3,64816 | 1,00E-26 | 2,40E-23 |
| Egr2 | ENSMUSG00000037868 | 3,02307 | 2,01E-23 | 3,60E-20 |
| Gm26870 | ENSMUSG00000097312 | 2,75911 | 7,47E-04 | 0,03169 |
| Igha | ENSMUSG00000095079 | 2,54849 | 1,34E-19 | 1,48E-16 |
| Gm10800 | ENSMUSG00000075014 | 2,5218 | 9,28E-04 | 0,03673 |
| Egr3 | ENSMUSG00000033730 | 2,45911 | 4,87E-11 | 3,17E-08 |
| Ptger4 | ENSMUSG00000039942 | 2,22599 | 1,80E-08 | 7,57E-06 |
| AI593442 | ENSMUSG00000078307 | 2,03939 | 2,09E-04 | 0,01355 |
| Fos | ENSMUSG00000021250 | 1,90006 | 6,33E-12 | 4,78E-09 |
| Jchain | ENSMUSG00000067149 | 1,8909 | 2,79E-06 | 4,75E-04 |
| Lyz1 | ENSMUSG00000069515 | 1,87149 | 0,00108 | 0,04102 |
| Igkc | ENSMUSG00000076609 | 1,84926 | 8,32E-05 | 0,00741 |
| Hcn1 | ENSMUSG00000021730 | 1,8414 | 0,00102 | 0,03912 |
| Dock2 | ENSMUSG00000020143 | 1,79337 | 2,86E-08 | 1,08E-05 |
| Apold1 | ENSMUSG00000090698 | 1,76125 | 7,23E-07 | 1,50E-04 |
| Rnf157 | ENSMUSG00000052949 | 1,75645 | 8,05E-19 | 8,24E-16 |
| Rmrp | ENSMUSG00000088088 | 1,66677 | 6,41E-07 | 1,39E-04 |
| Arc | ENSMUSG00000022602 | 1,65416 | 2,87E-07 | 7,91E-05 |
| Junb | ENSMUSG00000052837 | 1,62628 | 2,53E-20 | 3,30E-17 |
| Atp1a3 | ENSMUSG00000040907 | 1,58383 | 7,33E-04 | 0,03128 |
| Ier2 | ENSMUSG00000053560 | 1,54225 | 1,38E-20 | 1,98E-17 |
| Myc | ENSMUSG00000022346 | 1,51351 | 3,41E-11 | 2,33E-08 |
| Ckap2 | ENSMUSG00000037725 | 1,43815 | 0,00115 | 0,04288 |
| Rpph1 | ENSMUSG00000092837 | 1,37566 | 5,64E-07 | 1,26E-04 |
| Clec7a | ENSMUSG00000079293 | 1,35412 | 8,75E-06 | 0,00121 |
| Hoxa5 | ENSMUSG00000038253 | 1,34021 | 6,43E-04 | 0,02971 |
| Chac1 | ENSMUSG00000027313 | 1,21639 | 4,69E-08 | 1,53E-05 |
| Samd5 | ENSMUSG00000060487 | 1,19604 | 3,88E-05 | 0,00403 |
| Knstrn | ENSMUSG00000027331 | 1,18342 | 3,62E-04 | 0,01989 |
| Ccl7 | ENSMUSG00000035373 | 1,1829 | 0,00142 | 0,0492 |
| Zfp69 | ENSMUSG00000064141 | 1,11252 | 4,28E-04 | 0,02236 |
| Cldn5 | ENSMUSG00000041378 | 1,10341 | 2,64E-04 | 0,01599 |
| Cdk1 | ENSMUSG00000019942 | 1,0693 | 8,41E-04 | 0,03452 |
| Serpina3n | ENSMUSG00000021091 | 1,06159 | 7,97E-04 | 0,03328 |
| Sfn | ENSMUSG00000047281 | 1,04401 | 2,70E-04 | 0,01619 |
| Rrm2 | ENSMUSG00000020649 | 1,04089 | 0,00101 | 0,03895 |
| Frem2 | ENSMUSG00000037016 | 1,03862 | 6,29E-11 | 3,92E-08 |
| Epop | ENSMUSG00000043439 | 1,01255 | 5,50E-04 | 0,02662 |
| Gadd45b | ENSMUSG00000015312 | 1,0078 | 4,00E-07 | 1,02E-04 |
| Tcf15 | ENSMUSG00000068079 | 1,00349 | 3,49E-15 | 3,12E-12 |
| Dnaja1 | ENSMUSG00000028410 | -1,00098 | 5,59E-04 | 0,02687 |
| Banp | ENSMUSG00000025316 | -1,06359 | 1,20E-07 | 3,45E-05 |
| Dnm3os | ENSMUSG00000078190 | -1,07478 | 0,00124 | 0,04472 |
| Atcay | ENSMUSG00000034958 | -1,08479 | 9,84E-04 | 0,03833 |
| Ip6k3 | ENSMUSG00000024210 | -1,08827 | 5,14E-08 | 1,64E-05 |
| Dnajb1 | ENSMUSG00000005483 | -1,10265 | 2,67E-09 | 1,42E-06 |
| Nnmt | ENSMUSG00000032271 | -1,10983 | 1,11E-04 | 0,00869 |
| Rsrp1 | ENSMUSG00000037266 | -1,1234 | 4,15E-27 | 1,19E-23 |
| Gm31520 | ENSMUSG00000107653 | -1,13706 | 0,0013 | 0,04622 |
| Cdh20 | ENSMUSG00000050840 | -1,13841 | 7,43E-04 | 0,0316 |
| Trnp1 | ENSMUSG00000056596 | -1,14312 | 1,99E-10 | 1,19E-07 |
| Fam84b | ENSMUSG00000072568 | -1,1449 | 8,95E-08 | 2,73E-05 |
| Tnfrsf25 | ENSMUSG00000024793 | -1,18349 | 3,29E-06 | 5,48E-04 |
| Ppp1r3c | ENSMUSG00000067279 | -1,18423 | 6,51E-04 | 0,02992 |
| Dbh | ENSMUSG00000000889 | -1,20024 | 1,03E-04 | 0,00834 |
| Irs3 | ENSMUSG00000047182 | -1,23278 | 1,86E-04 | 0,01249 |
| Gm16023 | ENSMUSG00000086682 | -1,236 | 9,02E-04 | 0,03603 |
| Fam222a | ENSMUSG00000041930 | -1,25639 | 4,42E-07 | 1,09E-04 |
| Plekhh1 | ENSMUSG00000060716 | -1,25884 | 5,63E-07 | 1,26E-04 |
| 9430085M18Rik | ENSMUSG00000106164 | -1,27489 | 5,43E-04 | 0,02637 |
| Tagap | ENSMUSG00000033450 | -1,37533 | 6,71E-05 | 0,00613 |
| Gm43980 | ENSMUSG00000107962 | -1,39463 | 1,06E-05 | 0,00141 |
| BC018473 | ENSMUSG00000056032 | -1,44448 | 1,66E-06 | 3,04E-04 |
| Gm30238 | ENSMUSG00000103898 | -1,46648 | 5,75E-04 | 0,02736 |
| Pts | ENSMUSG00000032067 | -1,48659 | 8,99E-39 | 4,30E-35 |
| Rp1 | ENSMUSG00000025900 | -1,49819 | 1,09E-04 | 0,00857 |
| Sypl2 | ENSMUSG00000027887 | -1,52636 | 1,35E-04 | 0,01011 |
| 2610306O10Rik | ENSMUSG00000108614 | -1,59717 | 7,87E-04 | 0,03298 |
| Dixdc1 | ENSMUSG00000032064 | -1,61205 | 9,61E-20 | 1,15E-16 |
| Ccdc63 | ENSMUSG00000043036 | -1,65832 | 2,80E-04 | 0,01654 |
| Lpar3 | ENSMUSG00000036832 | -1,72077 | 1,96E-04 | 0,01294 |
| 1700119I11Rik | ENSMUSG00000082488 | -1,76529 | 2,38E-04 | 0,01489 |
| Noxred1 | ENSMUSG00000072919 | -1,95894 | 4,44E-04 | 0,02275 |
| Nxpe2 | ENSMUSG00000032028 | -1,98387 | 5,10E-07 | 1,18E-04 |
| Nrg4 | ENSMUSG00000032311 | -3,02495 | 3,90E-15 | 3,29E-12 |
| Hspa1a | ENSMUSG00000091971 | -3,12862 | 2,16E-05 | 0,00249 |
| Gm12416 | ENSMUSG00000083044 | -3,92415 | 3,83E-47 | 2,74E-43 |
| Hspa1b | ENSMUSG00000090877 | -4,09926 | 4,80E-07 | 1,15E-04 |
| Gm43305 | ENSMUSG00000105703 | -4,22103 | 0,00104 | 0,03983 |
| Nxpe4 | ENSMUSG00000044229 | -5,38439 | 4,09E-53 | 5,85E-49 |
| Gm10290 | ENSMUSG00000105775 | -5,43154 | 7,64E-25 | 1,56E-21 |
| Plet1os | ENSMUSG00000101304 | -6,35954 | 5,50E-33 | 1,97E-29 |

**Differentially expressed genes in the left ventricle (WT-Sham vs. WT-TAC)**

| Gen Symbol | Gen ID | log2FoldChange | p-value | Padj |
| --- | --- | --- | --- | --- |
| Igfbp2 | ENSMUSG00000039323 | 4,06915 | 7,70E-06 | 4,29E-04 |
| Egr2 | ENSMUSG00000037868 | 3,68606 | 1,48E-22 | 2,37E-19 |
| Egr1 | ENSMUSG00000038418 | 3,55498 | 2,11E-67 | 3,04E-63 |
| Igha | ENSMUSG00000095079 | 3,10603 | 3,96E-10 | 1,02E-07 |
| Slc15a2 | ENSMUSG00000022899 | 3,07477 | 6,97E-07 | 6,08E-05 |
| S100a9 | ENSMUSG00000056071 | 3,02645 | 2,93E-08 | 4,02E-06 |
| Acta1 | ENSMUSG00000031972 | 2,99804 | 4,03E-34 | 1,94E-30 |
| Egr3 | ENSMUSG00000033730 | 2,95399 | 4,37E-18 | 4,20E-15 |
| Hcn1 | ENSMUSG00000021730 | 2,5906 | 9,19E-07 | 7,53E-05 |
| Igkc | ENSMUSG00000076609 | 2,37819 | 1,87E-10 | 5,29E-08 |
| Jchain | ENSMUSG00000067149 | 2,37552 | 2,35E-05 | 0,00101 |
| Pdzd3 | ENSMUSG00000032105 | 2,34623 | 4,21E-05 | 0,00155 |
| Ppp1r3g | ENSMUSG00000050423 | 2,22024 | 9,51E-04 | 0,01595 |
| Fhad1 | ENSMUSG00000051435 | 2,20379 | 4,99E-04 | 0,00993 |
| Edn3 | ENSMUSG00000027524 | 2,16761 | 1,13E-16 | 8,58E-14 |
| St8sia1 | ENSMUSG00000030283 | 2,14338 | 1,92E-04 | 0,00491 |
| Sell | ENSMUSG00000026581 | 2,04933 | 4,97E-05 | 0,00177 |
| Fos | ENSMUSG00000021250 | 2,04643 | 1,25E-09 | 2,87E-07 |
| Gas2l3 | ENSMUSG00000074802 | 2,03812 | 3,00E-15 | 1,66E-12 |
| Lman1l | ENSMUSG00000056271 | 2,03654 | 2,94E-06 | 2,05E-04 |
| Myc | ENSMUSG00000022346 | 2,02167 | 1,72E-08 | 2,58E-06 |
| Ltbp2 | ENSMUSG00000002020 | 2,00367 | 2,49E-10 | 6,89E-08 |
| Tbx15 | ENSMUSG00000027868 | 1,97299 | 4,12E-07 | 3,89E-05 |
| Kcnq5 | ENSMUSG00000028033 | 1,9481 | 7,84E-05 | 0,00253 |
| Gm30873 | ENSMUSG00000109341 | 1,92321 | 1,03E-04 | 0,00313 |
| Crlf1 | ENSMUSG00000007888 | 1,89081 | 8,39E-07 | 7,16E-05 |
| Ngef | ENSMUSG00000026259 | 1,86334 | 2,46E-04 | 0,00596 |
| Junb | ENSMUSG00000052837 | 1,85335 | 6,22E-24 | 1,49E-20 |
| Dbp | ENSMUSG00000059824 | 1,81612 | 9,25E-04 | 0,01571 |
| Atp6v0a4 | ENSMUSG00000038600 | 1,77848 | 0,00343 | 0,0391 |
| Arc | ENSMUSG00000022602 | 1,7294 | 3,75E-08 | 4,84E-06 |
| Aqp8 | ENSMUSG00000030762 | 1,68775 | 1,18E-12 | 4,38E-10 |
| Met | ENSMUSG00000009376 | 1,67682 | 2,04E-05 | 9,10E-04 |
| Nlrc3 | ENSMUSG00000049871 | 1,66001 | 1,96E-08 | 2,83E-06 |
| Thbs1 | ENSMUSG00000040152 | 1,65969 | 6,35E-10 | 1,57E-07 |
| Serpine1 | ENSMUSG00000037411 | 1,63973 | 2,11E-04 | 0,00529 |
| Gm13943 | ENSMUSG00000085277 | 1,63285 | 1,20E-09 | 2,83E-07 |
| Cyr61 | ENSMUSG00000028195 | 1,62067 | 8,24E-28 | 2,38E-24 |
| Mybpc2 | ENSMUSG00000038670 | 1,61513 | 3,17E-21 | 4,16E-18 |
| Atp1a3 | ENSMUSG00000040907 | 1,5745 | 4,18E-04 | 0,00868 |
| Lrp8 | ENSMUSG00000028613 | 1,5729 | 1,32E-04 | 0,00369 |
| Kcnc1 | ENSMUSG00000058975 | 1,51614 | 2,28E-05 | 9,90E-04 |
| Hmmr | ENSMUSG00000020330 | 1,51224 | 0,00363 | 0,04062 |
| Nppa | ENSMUSG00000041616 | 1,48823 | 2,33E-04 | 0,00567 |
| Spsb4 | ENSMUSG00000046997 | 1,46958 | 0,00145 | 0,02162 |
| Klhl34 | ENSMUSG00000047485 | 1,44887 | 2,02E-11 | 6,62E-09 |
| Ptger4 | ENSMUSG00000039942 | 1,44479 | 3,56E-09 | 7,03E-07 |
| Sh3gl2 | ENSMUSG00000028488 | 1,43656 | 3,46E-06 | 2,30E-04 |
| Myot | ENSMUSG00000024471 | 1,42033 | 9,76E-08 | 1,11E-05 |
| Kntc1 | ENSMUSG00000029414 | 1,41592 | 0,00153 | 0,0223 |
| Synpo2l | ENSMUSG00000039376 | 1,40266 | 4,17E-14 | 1,77E-11 |
| Pamr1 | ENSMUSG00000027188 | 1,39328 | 1,58E-05 | 7,41E-04 |
| Mgat5b | ENSMUSG00000043857 | 1,38906 | 0,00208 | 0,02771 |
| Ksr2 | ENSMUSG00000025658 | 1,38756 | 1,20E-04 | 0,00345 |
| Myh7 | ENSMUSG00000053093 | 1,38585 | 4,09E-21 | 4,92E-18 |
| Ier2 | ENSMUSG00000053560 | 1,36189 | 8,64E-15 | 4,30E-12 |
| Tnfrsf12a | ENSMUSG00000023905 | 1,36016 | 1,20E-16 | 8,65E-14 |
| Cilp | ENSMUSG00000042254 | 1,35986 | 5,13E-08 | 6,37E-06 |
| Rnf165 | ENSMUSG00000025427 | 1,3596 | 2,69E-04 | 0,00638 |
| Hbegf | ENSMUSG00000024486 | 1,35911 | 2,23E-12 | 8,06E-10 |
| Col8a2 | ENSMUSG00000056174 | 1,35219 | 0,00274 | 0,03342 |
| Apold1 | ENSMUSG00000090698 | 1,34284 | 3,72E-07 | 3,57E-05 |
| Lingo1 | ENSMUSG00000049556 | 1,3316 | 0,00322 | 0,03741 |
| Col8a1 | ENSMUSG00000068196 | 1,27828 | 2,13E-37 | 1,54E-33 |
| Ankrd1 | ENSMUSG00000024803 | 1,26955 | 6,21E-23 | 1,12E-19 |
| Frem2 | ENSMUSG00000037016 | 1,2689 | 2,59E-06 | 1,85E-04 |
| Gadd45g | ENSMUSG00000021453 | 1,25856 | 1,34E-15 | 7,70E-13 |
| Lefty1 | ENSMUSG00000038793 | 1,25663 | 0,00116 | 0,01845 |
| Pou3f1 | ENSMUSG00000090125 | 1,25248 | 5,80E-04 | 0,01117 |
| Gdf15 | ENSMUSG00000038508 | 1,24199 | 6,71E-06 | 3,86E-04 |
| Fgf6 | ENSMUSG00000000183 | 1,23342 | 3,67E-04 | 0,00788 |
| Scd4 | ENSMUSG00000050195 | 1,23256 | 8,15E-05 | 0,0026 |
| Cxcl1 | ENSMUSG00000029380 | 1,21078 | 0,00215 | 0,02817 |
| Iqgap3 | ENSMUSG00000028068 | 1,19084 | 3,56E-04 | 0,00774 |
| Clec7a | ENSMUSG00000079293 | 1,18363 | 0,00167 | 0,02374 |
| Dkk3 | ENSMUSG00000030772 | 1,17697 | 1,18E-04 | 0,00338 |
| Tox3 | ENSMUSG00000043668 | 1,15747 | 8,76E-04 | 0,01507 |
| Xirp1 | ENSMUSG00000079243 | 1,14327 | 5,73E-09 | 1,06E-06 |
| Dock2 | ENSMUSG00000020143 | 1,13566 | 0,00316 | 0,03686 |
| Bhlhe41 | ENSMUSG00000030256 | 1,11761 | 4,49E-06 | 2,84E-04 |
| Dok7 | ENSMUSG00000044716 | 1,11573 | 1,46E-14 | 7,02E-12 |
| Gadd45b | ENSMUSG00000015312 | 1,11081 | 1,87E-05 | 8,43E-04 |
| Timp1 | ENSMUSG00000001131 | 1,10736 | 2,98E-04 | 0,00685 |
| Gm10129 | ENSMUSG00000103789 | 1,10713 | 5,89E-05 | 0,002 |
| Cdk1 | ENSMUSG00000019942 | 1,10274 | 0,00226 | 0,02927 |
| Wisp2 | ENSMUSG00000027656 | 1,09944 | 7,42E-04 | 0,01331 |
| Fstl4 | ENSMUSG00000036264 | 1,0919 | 3,85E-04 | 0,00815 |
| Chac1 | ENSMUSG00000027313 | 1,08775 | 6,79E-06 | 3,89E-04 |
| Cdc6 | ENSMUSG00000017499 | 1,08732 | 0,00327 | 0,03773 |
| Cpxm2 | ENSMUSG00000030862 | 1,07213 | 3,76E-08 | 4,84E-06 |
| Cnksr1 | ENSMUSG00000028841 | 1,06684 | 2,21E-09 | 4,68E-07 |
| Nox4 | ENSMUSG00000030562 | 1,06 | 0,0028 | 0,03378 |
| Bambi | ENSMUSG00000024232 | 1,04863 | 2,17E-11 | 6,95E-09 |
| Col12a1 | ENSMUSG00000032332 | 1,04753 | 0,0015 | 0,02209 |
| Ctgf | ENSMUSG00000019997 | 1,04254 | 0,00212 | 0,02799 |
| Abra | ENSMUSG00000042895 | 1,03821 | 2,82E-05 | 0,00116 |
| Dct | ENSMUSG00000022129 | 1,02606 | 2,19E-05 | 9,59E-04 |
| Shisa3 | ENSMUSG00000050010 | 1,02063 | 3,00E-05 | 0,00122 |
| Runx2 | ENSMUSG00000039153 | 1,02034 | 0,00426 | 0,04518 |
| Casq1 | ENSMUSG00000007122 | 1,01995 | 1,00E-04 | 0,00306 |
| Scml4 | ENSMUSG00000044770 | 1,01549 | 6,56E-05 | 0,0022 |
| Armcx4 | ENSMUSG00000049804 | 1,00829 | 2,34E-04 | 0,00569 |
| Tnc | ENSMUSG00000028364 | 1,00691 | 9,09E-06 | 4,93E-04 |
| Prepl | ENSMUSG00000024127 | 1,00632 | 1,30E-05 | 6,46E-04 |
| Per3 | ENSMUSG00000028957 | 1,00131 | 4,79E-04 | 0,0096 |
| Gm35339 | ENSMUSG00000109179 | -1,00212 | 0,00329 | 0,0379 |
| Gm17491 | ENSMUSG00000097042 | -1,00238 | 1,63E-04 | 0,00433 |
| Dnaaf3 | ENSMUSG00000055809 | -1,01265 | 3,67E-14 | 1,65E-11 |
| Gm16170 | ENSMUSG00000086245 | -1,01798 | 7,48E-04 | 0,01338 |
| Tmem56 | ENSMUSG00000028132 | -1,01857 | 3,75E-05 | 0,00145 |
| Crybb1 | ENSMUSG00000029343 | -1,01932 | 2,82E-04 | 0,00659 |
| Msc | ENSMUSG00000025930 | -1,03729 | 2,24E-04 | 0,00552 |
| Tgfbr3l | ENSMUSG00000089736 | -1,03772 | 0,00177 | 0,02467 |
| Celsr3 | ENSMUSG00000023473 | -1,05282 | 9,94E-06 | 5,19E-04 |
| 4930506C21Rik | ENSMUSG00000087478 | -1,05393 | 3,13E-04 | 0,00708 |
| Gm15903 | ENSMUSG00000086916 | -1,08356 | 0,0039 | 0,04269 |
| Gm16316 | ENSMUSG00000087129 | -1,096 | 8,60E-04 | 0,01483 |
| Acrbp | ENSMUSG00000072770 | -1,09667 | 0,00427 | 0,04518 |
| Chrm3 | ENSMUSG00000046159 | -1,09762 | 0,0023 | 0,02956 |
| Mir208b | ENSMUSG00000077928 | -1,10968 | 1,13E-06 | 8,92E-05 |
| Gm12258 | ENSMUSG00000072915 | -1,11918 | 1,49E-04 | 0,00404 |
| Sbk2 | ENSMUSG00000030433 | -1,12062 | 7,73E-05 | 0,00251 |
| Cngb3 | ENSMUSG00000056494 | -1,13347 | 5,53E-04 | 0,01078 |
| Frmpd3 | ENSMUSG00000042425 | -1,13441 | 5,75E-04 | 0,01111 |
| A330023F24Rik | ENSMUSG00000096929 | -1,14276 | 3,97E-05 | 0,00148 |
| Gm43359 | ENSMUSG00000105434 | -1,14523 | 8,31E-04 | 0,01453 |
| Aqp4 | ENSMUSG00000024411 | -1,15478 | 9,16E-05 | 0,00287 |
| Gm28979 | ENSMUSG00000101941 | -1,16242 | 2,61E-06 | 1,86E-04 |
| Klf2 | ENSMUSG00000055148 | -1,1716 | 5,58E-10 | 1,41E-07 |
| Cenpf | ENSMUSG00000026605 | -1,22308 | 0,00341 | 0,03903 |
| Olfr78 | ENSMUSG00000043366 | -1,22607 | 0,00177 | 0,02467 |
| 9430085M18Rik | ENSMUSG00000106164 | -1,22741 | 0,00148 | 0,02185 |
| Tbc1d10c | ENSMUSG00000040247 | -1,25562 | 2,31E-16 | 1,58E-13 |
| Slc22a1 | ENSMUSG00000023829 | -1,25852 | 9,52E-06 | 5,09E-04 |
| Gapdhs | ENSMUSG00000061099 | -1,27736 | 0,00377 | 0,04172 |
| Ccdc63 | ENSMUSG00000043036 | -1,28074 | 0,00129 | 0,01983 |
| Npas2 | ENSMUSG00000026077 | -1,29223 | 4,83E-04 | 0,00966 |
| Pla2g4b | ENSMUSG00000098488 | -1,29392 | 0,0018 | 0,02493 |
| Gm43360 | ENSMUSG00000105970 | -1,29766 | 0,00252 | 0,03158 |
| H19 | ENSMUSG00000000031 | -1,30337 | 0,00135 | 0,02045 |
| Rasl11a | ENSMUSG00000029641 | -1,30385 | 4,44E-06 | 2,82E-04 |
| Acot3 | ENSMUSG00000021228 | -1,3061 | 1,70E-04 | 0,00451 |
| Mas1 | ENSMUSG00000068037 | -1,31039 | 2,33E-04 | 0,00567 |
| Gm16023 | ENSMUSG00000086682 | -1,33094 | 3,07E-04 | 0,007 |
| Lgals4 | ENSMUSG00000053964 | -1,34855 | 2,60E-08 | 3,72E-06 |
| Rsrp1 | ENSMUSG00000037266 | -1,35438 | 4,05E-14 | 1,77E-11 |
| Fam84b | ENSMUSG00000072568 | -1,35839 | 4,52E-09 | 8,58E-07 |
| Atcay | ENSMUSG00000034958 | -1,36051 | 0,00226 | 0,02925 |
| Gm15893 | ENSMUSG00000085246 | -1,3608 | 0,00202 | 0,02709 |
| Gm10635 | ENSMUSG00000111765 | -1,36711 | 1,50E-05 | 7,18E-04 |
| Banf2os | ENSMUSG00000086384 | -1,36806 | 5,37E-07 | 4,93E-05 |
| Gm30238 | ENSMUSG00000103898 | -1,38602 | 0,00247 | 0,03117 |
| Rgn | ENSMUSG00000023070 | -1,39382 | 0,00295 | 0,03499 |
| Dbh | ENSMUSG00000000889 | -1,43579 | 7,81E-07 | 6,74E-05 |
| Irs3 | ENSMUSG00000047182 | -1,43641 | 2,27E-04 | 0,00557 |
| Gm45805 | ENSMUSG00000110626 | -1,46545 | 0,00237 | 0,03028 |
| Rp1 | ENSMUSG00000025900 | -1,47135 | 0,0011 | 0,01773 |
| Nrg2 | ENSMUSG00000060275 | -1,47416 | 9,78E-06 | 5,17E-04 |
| Cdh22 | ENSMUSG00000053166 | -1,48752 | 3,90E-09 | 7,61E-07 |
| Rps3a3 | ENSMUSG00000059751 | -1,49326 | 0,0021 | 0,02781 |
| Alkal2 | ENSMUSG00000054204 | -1,5182 | 1,27E-05 | 6,36E-04 |
| Gm20045 | ENSMUSG00000103983 | -1,52411 | 6,63E-07 | 5,83E-05 |
| 0610005C13Rik | ENSMUSG00000109644 | -1,53561 | 0,00108 | 0,0175 |
| Cacng6 | ENSMUSG00000078815 | -1,65694 | 3,75E-05 | 0,00145 |
| Cnmd | ENSMUSG00000022025 | -1,71337 | 1,01E-08 | 1,57E-06 |
| Gm43980 | ENSMUSG00000107962 | -1,79899 | 4,95E-09 | 9,26E-07 |
| 2610306O10Rik | ENSMUSG00000108614 | -1,84927 | 1,10E-04 | 0,00325 |
| Tmprss4 | ENSMUSG00000032091 | -2,0061 | 1,30E-05 | 6,46E-04 |
| Aldob | ENSMUSG00000028307 | -2,02946 | 7,57E-24 | 1,56E-20 |
| Dnase1l2 | ENSMUSG00000024136 | -2,09414 | 9,10E-07 | 7,50E-05 |
| Fgf10 | ENSMUSG00000021732 | -2,59522 | 6,99E-07 | 6,08E-05 |

**Differentially expressed genes in the left ventricle (HCN4F-Sham vs. HCN4F-TAC)**

| Gen Symbol | Gen ID | log2FoldChange | p-value | Padj |
| --- | --- | --- | --- | --- |
| Igfbp2 | ENSMUSG00000039323 | 6,10981 | 6,06E-08 | 4,28E-06 |
| Gm29773 | ENSMUSG00000110547 | 4,86301 | 4,02E-10 | 4,99E-08 |
| Ereg | ENSMUSG00000029377 | 4,84257 | 3,49E-07 | 1,92E-05 |
| Acta1 | ENSMUSG00000031972 | 4,24722 | 2,56E-45 | 1,82E-41 |
| Crlf1 | ENSMUSG00000007888 | 3,64627 | 9,72E-11 | 1,48E-08 |
| Vgll2 | ENSMUSG00000049641 | 3,46824 | 0,00253 | 0,02558 |
| Serpinb1c | ENSMUSG00000079049 | 3,38164 | 8,16E-10 | 9,46E-08 |
| Cilp | ENSMUSG00000042254 | 3,2737 | 3,31E-11 | 5,90E-09 |
| Nppa | ENSMUSG00000041616 | 3,21555 | 4,40E-10 | 5,41E-08 |
| Comp | ENSMUSG00000031849 | 3,19566 | 2,03E-07 | 1,21E-05 |
| Sprr1a | ENSMUSG00000050359 | 3,16329 | 2,22E-05 | 6,14E-04 |
| Has1 | ENSMUSG00000003665 | 3,14222 | 8,06E-07 | 3,90E-05 |
| Tbx15 | ENSMUSG00000027868 | 3,05168 | 4,78E-17 | 2,44E-14 |
| Fgf6 | ENSMUSG00000000183 | 3,03137 | 1,04E-05 | 3,35E-04 |
| Gm13054 | ENSMUSG00000086806 | 2,98898 | 2,32E-15 | 8,93E-13 |
| Ltbp2 | ENSMUSG00000002020 | 2,97302 | 1,38E-08 | 1,18E-06 |
| Lrp8 | ENSMUSG00000028613 | 2,96172 | 7,25E-14 | 2,11E-11 |
| Thbs4 | ENSMUSG00000021702 | 2,88119 | 8,48E-09 | 7,76E-07 |
| Lman1l | ENSMUSG00000056271 | 2,87668 | 2,81E-13 | 6,91E-11 |
| 1500015O10Rik | ENSMUSG00000026051 | 2,85411 | 6,81E-04 | 0,00958 |
| Fmod | ENSMUSG00000041559 | 2,83875 | 9,70E-04 | 0,01248 |
| Nlrc3 | ENSMUSG00000049871 | 2,79331 | 1,02E-20 | 9,68E-18 |
| Hspa1b | ENSMUSG00000090877 | 2,75425 | 2,11E-06 | 8,92E-05 |
| Aqp8 | ENSMUSG00000030762 | 2,74294 | 1,18E-22 | 1,91E-19 |
| Col8a2 | ENSMUSG00000056174 | 2,70084 | 1,01E-05 | 3,28E-04 |
| Atp6v0a4 | ENSMUSG00000038600 | 2,6693 | 3,07E-07 | 1,73E-05 |
| Tceal7 | ENSMUSG00000079428 | 2,62904 | 9,02E-08 | 5,90E-06 |
| Hspa1a | ENSMUSG00000091971 | 2,57726 | 1,48E-05 | 4,41E-04 |
| Grip1 | ENSMUSG00000034813 | 2,55913 | 8,73E-08 | 5,80E-06 |
| Mgat5b | ENSMUSG00000043857 | 2,55527 | 1,83E-12 | 3,96E-10 |
| Thbs1 | ENSMUSG00000040152 | 2,53698 | 7,72E-13 | 1,81E-10 |
| Frem1 | ENSMUSG00000059049 | 2,4948 | 9,29E-07 | 4,41E-05 |
| Edn3 | ENSMUSG00000027524 | 2,48731 | 2,92E-10 | 3,86E-08 |
| Cilp2 | ENSMUSG00000044006 | 2,47575 | 9,28E-05 | 0,00193 |
| Sypl2 | ENSMUSG00000027887 | 2,47221 | 2,02E-13 | 5,25E-11 |
| Ptgs2 | ENSMUSG00000032487 | 2,46661 | 1,82E-19 | 1,30E-16 |
| Myh7 | ENSMUSG00000053093 | 2,45885 | 1,77E-07 | 1,07E-05 |
| Syndig1 | ENSMUSG00000074736 | 2,42011 | 1,36E-06 | 6,11E-05 |
| Gpr176 | ENSMUSG00000040133 | 2,38465 | 1,16E-05 | 3,66E-04 |
| Prrx2 | ENSMUSG00000039476 | 2,33025 | 1,51E-04 | 0,0028 |
| Gdf15 | ENSMUSG00000038508 | 2,33014 | 8,48E-04 | 0,01134 |
| Synpo2l | ENSMUSG00000039376 | 2,3003 | 2,97E-48 | 4,24E-44 |
| Col9a2 | ENSMUSG00000028626 | 2,28966 | 4,85E-05 | 0,00112 |
| Ankrd1 | ENSMUSG00000024803 | 2,27776 | 9,61E-31 | 3,43E-27 |
| Itih2 | ENSMUSG00000037254 | 2,26978 | 2,47E-06 | 1,02E-04 |
| Nppb | ENSMUSG00000029019 | 2,26408 | 2,58E-07 | 1,49E-05 |
| Egr2 | ENSMUSG00000037868 | 2,22931 | 1,80E-06 | 7,82E-05 |
| Abra | ENSMUSG00000042895 | 2,1225 | 2,15E-06 | 9,08E-05 |
| St8sia1 | ENSMUSG00000030283 | 2,11481 | 5,40E-06 | 1,97E-04 |
| Spp1 | ENSMUSG00000029304 | 2,10629 | 1,07E-04 | 0,00214 |
| Slc24a2 | ENSMUSG00000037996 | 2,10329 | 7,18E-10 | 8,61E-08 |
| Kcnj14 | ENSMUSG00000058743 | 2,10182 | 4,38E-07 | 2,33E-05 |
| Dkk3 | ENSMUSG00000030772 | 2,09981 | 2,39E-04 | 0,00405 |
| Postn | ENSMUSG00000027750 | 2,07345 | 3,64E-07 | 1,98E-05 |
| Atf3 | ENSMUSG00000026628 | 2,04378 | 4,16E-04 | 0,00646 |
| Pamr1 | ENSMUSG00000027188 | 2,0414 | 1,51E-08 | 1,26E-06 |
| Scml4 | ENSMUSG00000044770 | 2,03302 | 9,56E-18 | 5,25E-15 |
| 5430419D17Rik | ENSMUSG00000006204 | 2,01228 | 3,91E-05 | 9,66E-04 |
| Fhad1 | ENSMUSG00000051435 | 2,01204 | 1,88E-04 | 0,00336 |
| Serpine1 | ENSMUSG00000037411 | 2,00359 | 4,98E-08 | 3,67E-06 |
| Adamtsl2 | ENSMUSG00000036040 | 1,98889 | 3,03E-09 | 3,13E-07 |
| Col12a1 | ENSMUSG00000032332 | 1,98133 | 3,24E-09 | 3,33E-07 |
| Fam167a | ENSMUSG00000035095 | 1,9655 | 8,36E-05 | 0,00178 |
| Sfrp2 | ENSMUSG00000027996 | 1,96371 | 3,37E-11 | 5,94E-09 |
| Ankrd2 | ENSMUSG00000025172 | 1,96326 | 2,05E-05 | 5,77E-04 |
| Krt18 | ENSMUSG00000023043 | 1,91404 | 5,65E-04 | 0,00824 |
| Ctgf | ENSMUSG00000019997 | 1,90195 | 4,67E-10 | 5,70E-08 |
| Ngef | ENSMUSG00000026259 | 1,87303 | 8,85E-07 | 4,25E-05 |
| Rcan1 | ENSMUSG00000022951 | 1,85718 | 1,19E-05 | 3,73E-04 |
| Gm30873 | ENSMUSG00000109341 | 1,85487 | 2,49E-05 | 6,71E-04 |
| Mfap4 | ENSMUSG00000042436 | 1,85207 | 1,94E-05 | 5,48E-04 |
| AW551984 | ENSMUSG00000038112 | 1,84168 | 2,42E-04 | 0,00409 |
| Col8a1 | ENSMUSG00000068196 | 1,83425 | 6,75E-21 | 6,88E-18 |
| Frzb | ENSMUSG00000027004 | 1,82797 | 4,66E-14 | 1,39E-11 |
| Ankrd23 | ENSMUSG00000067653 | 1,80261 | 3,24E-08 | 2,53E-06 |
| Gck | ENSMUSG00000041798 | 1,78592 | 1,56E-25 | 4,45E-22 |
| Hbegf | ENSMUSG00000024486 | 1,78304 | 6,12E-15 | 2,13E-12 |
| Nmrk2 | ENSMUSG00000004939 | 1,77616 | 3,51E-11 | 6,11E-09 |
| Dio2 | ENSMUSG00000007682 | 1,75712 | 2,37E-07 | 1,39E-05 |
| Egr3 | ENSMUSG00000033730 | 1,75385 | 1,22E-07 | 7,73E-06 |
| Xirp2 | ENSMUSG00000027022 | 1,732 | 2,39E-40 | 1,14E-36 |
| Itgbl1 | ENSMUSG00000032925 | 1,71507 | 1,80E-17 | 9,53E-15 |
| Padi4 | ENSMUSG00000025330 | 1,71189 | 9,17E-04 | 0,01199 |
| Car9 | ENSMUSG00000028463 | 1,7035 | 0,00362 | 0,03361 |
| Uck2 | ENSMUSG00000026558 | 1,69681 | 3,08E-19 | 2,00E-16 |
| Tnfrsf12a | ENSMUSG00000023905 | 1,6827 | 3,03E-19 | 2,00E-16 |
| F2rl1 | ENSMUSG00000021678 | 1,67314 | 5,75E-07 | 2,94E-05 |
| Ddias | ENSMUSG00000030641 | 1,65535 | 2,65E-04 | 0,00443 |
| Il21r | ENSMUSG00000030745 | 1,65028 | 2,22E-04 | 0,00383 |
| Tnc | ENSMUSG00000028364 | 1,63407 | 7,18E-09 | 6,66E-07 |
| Klhl34 | ENSMUSG00000047485 | 1,62923 | 7,26E-16 | 2,88E-13 |
| Timp1 | ENSMUSG00000001131 | 1,62544 | 0,00268 | 0,02673 |
| Pou3f1 | ENSMUSG00000090125 | 1,62285 | 1,12E-05 | 3,57E-04 |
| Cnksr1 | ENSMUSG00000028841 | 1,59586 | 7,11E-22 | 8,45E-19 |
| Otud1 | ENSMUSG00000043415 | 1,58658 | 4,69E-05 | 0,0011 |
| Gm12092 | ENSMUSG00000084850 | 1,58313 | 3,09E-05 | 8,04E-04 |
| Etv4 | ENSMUSG00000017724 | 1,58133 | 1,09E-04 | 0,00219 |
| Gm13943 | ENSMUSG00000085277 | 1,58088 | 2,30E-08 | 1,84E-06 |
| Fbln7 | ENSMUSG00000027386 | 1,58051 | 8,50E-04 | 0,01134 |
| Acan | ENSMUSG00000030607 | 1,56376 | 1,86E-04 | 0,00334 |
| Mfap5 | ENSMUSG00000030116 | 1,56364 | 2,46E-11 | 4,50E-09 |
| Mgam | ENSMUSG00000068587 | 1,55471 | 0,00305 | 0,02947 |
| Slc1a2 | ENSMUSG00000005089 | 1,55132 | 6,54E-04 | 0,00924 |
| Ccl12 | ENSMUSG00000035352 | 1,53213 | 9,61E-04 | 0,01242 |
| Cd79a | ENSMUSG00000003379 | 1,50082 | 4,83E-05 | 0,00112 |
| Xirp1 | ENSMUSG00000079243 | 1,49638 | 1,20E-06 | 5,51E-05 |
| Enah | ENSMUSG00000022995 | 1,49579 | 4,78E-20 | 4,27E-17 |
| Nox4 | ENSMUSG00000030562 | 1,49312 | 1,24E-05 | 3,86E-04 |
| Shisa3 | ENSMUSG00000050010 | 1,4919 | 1,03E-05 | 3,32E-04 |
| Adamts8 | ENSMUSG00000031994 | 1,48162 | 6,63E-07 | 3,31E-05 |
| Mybpc2 | ENSMUSG00000038670 | 1,47772 | 6,53E-15 | 2,22E-12 |
| Cpxm2 | ENSMUSG00000030862 | 1,47278 | 1,97E-12 | 4,13E-10 |
| Psma8 | ENSMUSG00000036743 | 1,469 | 0,00167 | 0,01894 |
| Gas2l3 | ENSMUSG00000074802 | 1,45943 | 1,59E-06 | 6,97E-05 |
| Met | ENSMUSG00000009376 | 1,44934 | 1,48E-09 | 1,65E-07 |
| Jsrp1 | ENSMUSG00000020216 | 1,44054 | 1,14E-04 | 0,00226 |
| Meox1 | ENSMUSG00000001493 | 1,43682 | 1,58E-10 | 2,19E-08 |
| Clec11a | ENSMUSG00000004473 | 1,43661 | 2,80E-04 | 0,00465 |
| Sox9 | ENSMUSG00000000567 | 1,43332 | 2,74E-14 | 8,51E-12 |
| Emp1 | ENSMUSG00000030208 | 1,42845 | 1,92E-13 | 5,12E-11 |
| Piezo2 | ENSMUSG00000041482 | 1,42263 | 7,85E-04 | 0,01071 |
| Ankrd45 | ENSMUSG00000044835 | 1,39938 | 2,70E-07 | 1,55E-05 |
| Nuak1 | ENSMUSG00000020032 | 1,39598 | 8,11E-14 | 2,32E-11 |
| Fibin | ENSMUSG00000074971 | 1,39014 | 8,73E-08 | 5,80E-06 |
| Krt80 | ENSMUSG00000037185 | 1,38979 | 1,02E-09 | 1,17E-07 |
| Vcan | ENSMUSG00000021614 | 1,3875 | 2,40E-25 | 5,71E-22 |
| Hectd2os | ENSMUSG00000087579 | 1,38034 | 7,78E-18 | 4,44E-15 |
| 1500009L16Rik | ENSMUSG00000087651 | 1,37138 | 3,10E-08 | 2,43E-06 |
| Klhl29 | ENSMUSG00000020627 | 1,36967 | 1,21E-06 | 5,52E-05 |
| Col4a3 | ENSMUSG00000079465 | 1,36359 | 3,03E-13 | 7,33E-11 |
| Gdf6 | ENSMUSG00000051279 | 1,36305 | 3,28E-05 | 8,43E-04 |
| Wisp2 | ENSMUSG00000027656 | 1,35769 | 4,83E-08 | 3,60E-06 |
| Panx1 | ENSMUSG00000031934 | 1,32501 | 9,05E-09 | 8,02E-07 |
| Hspa1l | ENSMUSG00000007033 | 1,32485 | 6,71E-12 | 1,33E-09 |
| Fhl1 | ENSMUSG00000023092 | 1,31414 | 1,13E-14 | 3,66E-12 |
| Dclk3 | ENSMUSG00000032500 | 1,30213 | 0,00501 | 0,0426 |
| Myot | ENSMUSG00000024471 | 1,29293 | 7,37E-09 | 6,79E-07 |
| Ptx3 | ENSMUSG00000027832 | 1,28922 | 0,00564 | 0,04662 |
| Inhba | ENSMUSG00000041324 | 1,28363 | 6,70E-06 | 2,35E-04 |
| Socs3 | ENSMUSG00000053113 | 1,27959 | 1,65E-06 | 7,20E-05 |
| Ppp1r3c | ENSMUSG00000067279 | 1,27878 | 0,00169 | 0,01907 |
| Bgn | ENSMUSG00000031375 | 1,27662 | 4,49E-11 | 7,63E-09 |
| Arhgdig | ENSMUSG00000073433 | 1,26794 | 0,00131 | 0,01589 |
| Ighd | ENSMUSG00000104213 | 1,26592 | 0,00324 | 0,03099 |
| Adamts20 | ENSMUSG00000022449 | 1,26397 | 0,00423 | 0,03774 |
| Gm31678 | ENSMUSG00000105427 | 1,25028 | 0,00159 | 0,0183 |
| Gnao1 | ENSMUSG00000031748 | 1,24845 | 2,47E-18 | 1,53E-15 |
| Ch25h | ENSMUSG00000050370 | 1,24259 | 0,00414 | 0,03726 |
| Fstl3 | ENSMUSG00000020325 | 1,23888 | 1,23E-10 | 1,81E-08 |
| Col14a1 | ENSMUSG00000022371 | 1,23246 | 1,77E-07 | 1,07E-05 |
| Fgl2 | ENSMUSG00000039899 | 1,23163 | 1,03E-10 | 1,55E-08 |
| Cyr61 | ENSMUSG00000028195 | 1,23019 | 4,41E-18 | 2,62E-15 |
| Tgfb2 | ENSMUSG00000039239 | 1,22083 | 1,27E-11 | 2,44E-09 |
| Atp8a2 | ENSMUSG00000021983 | 1,21558 | 1,11E-08 | 9,57E-07 |
| Svep1 | ENSMUSG00000028369 | 1,21425 | 9,14E-09 | 8,05E-07 |
| Sphk1 | ENSMUSG00000061878 | 1,20578 | 2,21E-06 | 9,26E-05 |
| Lox | ENSMUSG00000024529 | 1,20219 | 8,86E-09 | 7,98E-07 |
| Apod | ENSMUSG00000022548 | 1,20072 | 7,61E-08 | 5,20E-06 |
| Clcnkb | ENSMUSG00000006216 | 1,1994 | 1,80E-04 | 0,00325 |
| Nupr1 | ENSMUSG00000030717 | 1,19889 | 5,38E-05 | 0,00123 |
| Bcl2 | ENSMUSG00000057329 | 1,194 | 1,76E-08 | 1,43E-06 |
| Spsb4 | ENSMUSG00000046997 | 1,19039 | 2,03E-16 | 9,64E-14 |
| Adcy7 | ENSMUSG00000031659 | 1,18467 | 1,32E-11 | 2,52E-09 |
| Enpp1 | ENSMUSG00000037370 | 1,1809 | 1,13E-06 | 5,21E-05 |
| Hmmr | ENSMUSG00000020330 | 1,17651 | 0,00415 | 0,03733 |
| Mllt11 | ENSMUSG00000053192 | 1,17477 | 5,92E-16 | 2,49E-13 |
| Rab27b | ENSMUSG00000024511 | 1,17089 | 9,09E-05 | 0,0019 |
| Pi16 | ENSMUSG00000024011 | 1,17068 | 1,53E-07 | 9,40E-06 |
| AI839979 | ENSMUSG00000107355 | 1,16753 | 2,91E-06 | 1,18E-04 |
| Csf2rb2 | ENSMUSG00000071714 | 1,16455 | 6,08E-04 | 0,00874 |
| Syt12 | ENSMUSG00000049303 | 1,15508 | 3,34E-06 | 1,31E-04 |
| Col1a1 | ENSMUSG00000001506 | 1,14302 | 8,83E-09 | 7,98E-07 |
| Kif1a | ENSMUSG00000014602 | 1,13939 | 7,62E-04 | 0,01044 |
| Pfkp | ENSMUSG00000021196 | 1,13732 | 1,94E-13 | 5,12E-11 |
| Col22a1 | ENSMUSG00000079022 | 1,13457 | 0,00149 | 0,01756 |
| Rasl11b | ENSMUSG00000049907 | 1,13219 | 7,88E-04 | 0,01074 |
| Mmp3 | ENSMUSG00000043613 | 1,12187 | 1,23E-06 | 5,57E-05 |
| Ccl2 | ENSMUSG00000035385 | 1,12135 | 1,80E-05 | 5,17E-04 |
| Loxl2 | ENSMUSG00000034205 | 1,11012 | 4,01E-10 | 4,99E-08 |
| Itga9 | ENSMUSG00000039115 | 1,10761 | 7,01E-16 | 2,86E-13 |
| Col5a2 | ENSMUSG00000026042 | 1,10731 | 4,29E-12 | 8,88E-10 |
| Cd44 | ENSMUSG00000005087 | 1,10112 | 4,95E-09 | 4,77E-07 |
| Napepld | ENSMUSG00000044968 | 1,09891 | 2,77E-13 | 6,91E-11 |
| Lrp11 | ENSMUSG00000019796 | 1,09797 | 6,43E-06 | 2,27E-04 |
| Tmem119 | ENSMUSG00000054675 | 1,09251 | 2,09E-04 | 0,00365 |
| Tlr4 | ENSMUSG00000039005 | 1,08766 | 5,79E-09 | 5,55E-07 |
| Serpinf1 | ENSMUSG00000000753 | 1,08649 | 2,02E-07 | 1,21E-05 |
| Mthfd2 | ENSMUSG00000005667 | 1,08314 | 1,92E-08 | 1,55E-06 |
| Mapk4 | ENSMUSG00000024558 | 1,08106 | 3,74E-06 | 1,44E-04 |
| Pdgfrl | ENSMUSG00000031595 | 1,06978 | 2,99E-04 | 0,0049 |
| Dbn1 | ENSMUSG00000034675 | 1,06856 | 1,06E-08 | 9,22E-07 |
| Snai1 | ENSMUSG00000042821 | 1,06773 | 3,86E-05 | 9,57E-04 |
| Gm11967 | ENSMUSG00000084819 | 1,06131 | 2,36E-04 | 0,00401 |
| Scx | ENSMUSG00000034161 | 1,06106 | 1,41E-04 | 0,00268 |
| Ssc5d | ENSMUSG00000035279 | 1,06038 | 6,96E-07 | 3,45E-05 |
| Fam26e | ENSMUSG00000049872 | 1,0598 | 3,43E-07 | 1,89E-05 |
| Eln | ENSMUSG00000029675 | 1,05872 | 0,00221 | 0,02334 |
| Fstl1 | ENSMUSG00000022816 | 1,05731 | 5,60E-11 | 9,19E-09 |
| P3h2 | ENSMUSG00000038168 | 1,0481 | 9,77E-09 | 8,55E-07 |
| Kif26b | ENSMUSG00000026494 | 1,04668 | 5,69E-05 | 0,00129 |
| Nr4a1 | ENSMUSG00000023034 | 1,045 | 0,0013 | 0,01579 |
| Ccdc136 | ENSMUSG00000029769 | 1,04429 | 3,22E-06 | 1,27E-04 |
| Slc1a4 | ENSMUSG00000020142 | 1,04126 | 1,21E-06 | 5,51E-05 |
| Flnc | ENSMUSG00000068699 | 1,03254 | 1,78E-19 | 1,30E-16 |
| Sorbs2 | ENSMUSG00000031626 | 1,02782 | 2,48E-15 | 9,32E-13 |
| Gpr68 | ENSMUSG00000047415 | 1,02734 | 0,00579 | 0,0474 |
| Runx1 | ENSMUSG00000022952 | 1,02645 | 2,66E-05 | 7,03E-04 |
| Ptn | ENSMUSG00000029838 | 1,02258 | 0,00158 | 0,01823 |
| Bhlhe40 | ENSMUSG00000030103 | 1,02226 | 4,00E-06 | 1,53E-04 |
| Adam12 | ENSMUSG00000054555 | 1,02082 | 9,89E-04 | 0,01265 |
| Aldh1a2 | ENSMUSG00000013584 | 1,01957 | 8,95E-06 | 2,96E-04 |
| Sh3rf3 | ENSMUSG00000037990 | 1,01012 | 2,52E-04 | 0,00424 |
| Nrap | ENSMUSG00000049134 | 1,00606 | 5,10E-20 | 4,28E-17 |
| Esm1 | ENSMUSG00000042379 | 1,00447 | 6,95E-04 | 0,00971 |
| Tspan9 | ENSMUSG00000030352 | 1,0038 | 5,76E-15 | 2,05E-12 |
| Col3a1 | ENSMUSG00000026043 | 1,00365 | 2,17E-07 | 1,28E-05 |
| Banf2os | ENSMUSG00000086384 | -1,00106 | 0,00129 | 0,01577 |
| Slc35f1 | ENSMUSG00000038602 | -1,00166 | 0,00232 | 0,02411 |
| Abca12 | ENSMUSG00000050296 | -1,00175 | 6,09E-05 | 0,00136 |
| Gm37621 | ENSMUSG00000103672 | -1,00853 | 0,00408 | 0,03691 |
| Chrna10 | ENSMUSG00000066279 | -1,00948 | 1,89E-04 | 0,00336 |
| Epb41l4b | ENSMUSG00000028434 | -1,00989 | 1,03E-05 | 3,32E-04 |
| Ptpru | ENSMUSG00000028909 | -1,01362 | 4,21E-06 | 1,59E-04 |
| Tnip3 | ENSMUSG00000044162 | -1,01558 | 0,00146 | 0,01727 |
| Gstt2 | ENSMUSG00000033318 | -1,01786 | 6,07E-04 | 0,00874 |
| Gnmt | ENSMUSG00000002769 | -1,01926 | 0,00204 | 0,02193 |
| Tesmin | ENSMUSG00000024905 | -1,02089 | 0,00296 | 0,02878 |
| Foxo6 | ENSMUSG00000052135 | -1,02103 | 1,37E-05 | 4,16E-04 |
| Gm11816 | ENSMUSG00000086233 | -1,02877 | 0,00301 | 0,0292 |
| Slc6a17 | ENSMUSG00000027894 | -1,03002 | 4,42E-06 | 1,65E-04 |
| Plekhb1 | ENSMUSG00000030701 | -1,03759 | 3,07E-06 | 1,23E-04 |
| Clec18a | ENSMUSG00000033633 | -1,03907 | 0,00443 | 0,03903 |
| Sox10 | ENSMUSG00000033006 | -1,0395 | 0,00102 | 0,01292 |
| Pla2g5 | ENSMUSG00000041193 | -1,05172 | 4,23E-06 | 1,59E-04 |
| Arrdc2 | ENSMUSG00000002910 | -1,05615 | 2,04E-09 | 2,18E-07 |
| A330023F24Rik | ENSMUSG00000096929 | -1,0577 | 0,00286 | 0,02801 |
| Ddit4l | ENSMUSG00000046818 | -1,05947 | 0,00233 | 0,02413 |
| Ramp1 | ENSMUSG00000034353 | -1,05979 | 5,92E-07 | 3,01E-05 |
| Mbp | ENSMUSG00000041607 | -1,07497 | 1,20E-05 | 3,76E-04 |
| Tmem179 | ENSMUSG00000054013 | -1,07539 | 4,85E-05 | 0,00112 |
| Cryba4 | ENSMUSG00000066975 | -1,07784 | 8,30E-05 | 0,00177 |
| Gpr160 | ENSMUSG00000037661 | -1,09389 | 6,45E-05 | 0,00143 |
| Angptl3 | ENSMUSG00000028553 | -1,10772 | 0,00281 | 0,02757 |
| Frat1 | ENSMUSG00000067199 | -1,11029 | 9,31E-07 | 4,41E-05 |
| Gpr37l1 | ENSMUSG00000026424 | -1,11136 | 4,09E-04 | 0,00637 |
| Gal3st2c | ENSMUSG00000073608 | -1,11212 | 1,06E-06 | 4,93E-05 |
| Stard10 | ENSMUSG00000030688 | -1,12347 | 1,66E-10 | 2,28E-08 |
| Gm43359 | ENSMUSG00000105434 | -1,12726 | 9,65E-04 | 0,01246 |
| Pdp2 | ENSMUSG00000048371 | -1,13819 | 2,46E-09 | 2,59E-07 |
| Plxnb3 | ENSMUSG00000031385 | -1,13911 | 2,59E-06 | 1,06E-04 |
| Adam23 | ENSMUSG00000025964 | -1,1394 | 9,97E-05 | 0,00204 |
| Enpp2 | ENSMUSG00000022425 | -1,14138 | 2,63E-05 | 6,99E-04 |
| Moxd1 | ENSMUSG00000020000 | -1,14579 | 9,73E-04 | 0,01251 |
| Gm37033 | ENSMUSG00000104388 | -1,148 | 0,00327 | 0,03122 |
| Ptgfr | ENSMUSG00000028036 | -1,15146 | 4,20E-04 | 0,00651 |
| Gm27202 | ENSMUSG00000098424 | -1,15945 | 0,00567 | 0,04676 |
| Abca4 | ENSMUSG00000028125 | -1,16382 | 1,88E-06 | 8,10E-05 |
| Gm28979 | ENSMUSG00000101941 | -1,18163 | 0,0014 | 0,01669 |
| Stum | ENSMUSG00000053963 | -1,18253 | 2,29E-04 | 0,00394 |
| Aldh1l2 | ENSMUSG00000020256 | -1,19818 | 6,99E-05 | 0,00152 |
| Foxd3 | ENSMUSG00000067261 | -1,20149 | 1,65E-04 | 0,00303 |
| Gfra4 | ENSMUSG00000027316 | -1,20897 | 1,01E-09 | 1,16E-07 |
| Tnni2 | ENSMUSG00000031097 | -1,21245 | 0,00106 | 0,01339 |
| Ctla4 | ENSMUSG00000026011 | -1,22243 | 0,0035 | 0,03295 |
| Lvrn | ENSMUSG00000024481 | -1,2305 | 9,94E-05 | 0,00204 |
| Gm10435 | ENSMUSG00000072902 | -1,23896 | 1,48E-07 | 9,12E-06 |
| Hnmt | ENSMUSG00000026986 | -1,24088 | 3,88E-11 | 6,67E-09 |
| Cfap74 | ENSMUSG00000078490 | -1,24222 | 0,00117 | 0,01466 |
| Scara5 | ENSMUSG00000022032 | -1,24243 | 4,46E-08 | 3,37E-06 |
| Kcna6 | ENSMUSG00000038077 | -1,24436 | 0,0013 | 0,01585 |
| Adcy8 | ENSMUSG00000022376 | -1,24917 | 0,00316 | 0,03029 |
| Celsr3 | ENSMUSG00000023473 | -1,25134 | 1,67E-05 | 4,87E-04 |
| Gm42517 | ENSMUSG00000105867 | -1,25434 | 8,80E-04 | 0,01167 |
| Chl1 | ENSMUSG00000030077 | -1,25485 | 7,01E-04 | 0,00977 |
| Sorcs2 | ENSMUSG00000029093 | -1,25752 | 3,12E-16 | 1,44E-13 |
| Osr1 | ENSMUSG00000048387 | -1,25886 | 5,46E-05 | 0,00125 |
| Phkg1 | ENSMUSG00000025537 | -1,25983 | 1,15E-07 | 7,34E-06 |
| Gata3 | ENSMUSG00000015619 | -1,26658 | 0,00456 | 0,0398 |
| Epop | ENSMUSG00000043439 | -1,2675 | 4,29E-04 | 0,0066 |
| mt-Tt | ENSMUSG00000064371 | -1,27417 | 5,66E-08 | 4,02E-06 |
| Gm37320 | ENSMUSG00000104324 | -1,27531 | 0,00398 | 0,03624 |
| Snai3 | ENSMUSG00000006587 | -1,27825 | 1,65E-04 | 0,00303 |
| Dbh | ENSMUSG00000000889 | -1,28071 | 0,00283 | 0,02779 |
| 2210407C18Rik | ENSMUSG00000037145 | -1,28544 | 4,15E-06 | 1,58E-04 |
| Col6a6 | ENSMUSG00000043719 | -1,28835 | 6,05E-06 | 2,16E-04 |
| Opn4 | ENSMUSG00000021799 | -1,2975 | 7,97E-08 | 5,41E-06 |
| Lncpint | ENSMUSG00000044471 | -1,3124 | 0,00203 | 0,02185 |
| Chrna2 | ENSMUSG00000022041 | -1,32045 | 2,15E-04 | 0,00374 |
| Gm12519 | ENSMUSG00000085643 | -1,32581 | 6,85E-04 | 0,00962 |
| NA | ENSMUSG00000109908 | -1,3273 | 0,00114 | 0,01424 |
| Lypd2 | ENSMUSG00000022595 | -1,33095 | 0,00549 | 0,04558 |
| A530016L24Rik | ENSMUSG00000043122 | -1,3329 | 9,53E-15 | 3,16E-12 |
| Cenpf | ENSMUSG00000026605 | -1,33871 | 6,35E-13 | 1,51E-10 |
| Ccbe1 | ENSMUSG00000046318 | -1,35052 | 2,04E-06 | 8,67E-05 |
| Mir208b | ENSMUSG00000077928 | -1,3559 | 5,19E-05 | 0,00119 |
| Iglon5 | ENSMUSG00000013367 | -1,3587 | 0,00127 | 0,01553 |
| Ly75 | ENSMUSG00000026980 | -1,36995 | 2,53E-05 | 6,81E-04 |
| Cngb3 | ENSMUSG00000056494 | -1,37391 | 5,72E-04 | 0,0083 |
| Clcn1 | ENSMUSG00000029862 | -1,38094 | 6,89E-05 | 0,00151 |
| Cma1 | ENSMUSG00000022225 | -1,3841 | 0,001 | 0,01277 |
| Scarna17 | ENSMUSG00000088689 | -1,4187 | 4,60E-04 | 0,00695 |
| Plp1 | ENSMUSG00000031425 | -1,42224 | 7,70E-07 | 3,78E-05 |
| Cnmd | ENSMUSG00000022025 | -1,42751 | 0,00473 | 0,04085 |
| Drd2 | ENSMUSG00000032259 | -1,42784 | 5,04E-05 | 0,00116 |
| Gm2115 | ENSMUSG00000097789 | -1,45254 | 0,00478 | 0,04108 |
| Ano10 | ENSMUSG00000037949 | -1,46272 | 1,20E-22 | 1,91E-19 |
| Slc22a1 | ENSMUSG00000023829 | -1,47471 | 1,95E-05 | 5,50E-04 |
| Hrk | ENSMUSG00000046607 | -1,47589 | 0,00146 | 0,01728 |
| Sema4f | ENSMUSG00000000627 | -1,47877 | 0,0036 | 0,03349 |
| Scn10a | ENSMUSG00000034533 | -1,4946 | 1,70E-04 | 0,0031 |
| Gm33543 | ENSMUSG00000110353 | -1,49956 | 4,51E-06 | 1,68E-04 |
| Tmprss4 | ENSMUSG00000032091 | -1,50153 | 8,55E-05 | 0,00181 |
| Cfap61 | ENSMUSG00000037143 | -1,52585 | 0,00211 | 0,02254 |
| Cdh22 | ENSMUSG00000053166 | -1,53522 | 1,02E-04 | 0,00208 |
| Gm4956 | ENSMUSG00000025936 | -1,53906 | 6,48E-04 | 0,00917 |
| Kcna1 | ENSMUSG00000047976 | -1,53934 | 4,60E-05 | 0,00109 |
| Acot3 | ENSMUSG00000021228 | -1,54237 | 0,0011 | 0,01384 |
| S1pr5 | ENSMUSG00000045087 | -1,54376 | 8,28E-04 | 0,01115 |
| Lgals4 | ENSMUSG00000053964 | -1,55171 | 1,88E-10 | 2,55E-08 |
| Ces1d | ENSMUSG00000056973 | -1,55236 | 4,05E-05 | 9,89E-04 |
| Gm44022 | ENSMUSG00000107706 | -1,56704 | 0,00301 | 0,02916 |
| Gfra3 | ENSMUSG00000024366 | -1,57333 | 1,44E-05 | 4,31E-04 |
| Nmb | ENSMUSG00000025723 | -1,58529 | 9,90E-08 | 6,39E-06 |
| Acsm5 | ENSMUSG00000030972 | -1,62226 | 1,64E-07 | 1,00E-05 |
| Gm42639 | ENSMUSG00000104871 | -1,62874 | 0,00406 | 0,03672 |
| 2010001K21Rik | ENSMUSG00000051606 | -1,63621 | 0,00311 | 0,02991 |
| Tbc1d10c | ENSMUSG00000040247 | -1,65125 | 3,12E-14 | 9,48E-12 |
| Sbk2 | ENSMUSG00000030433 | -1,65504 | 1,74E-06 | 7,59E-05 |
| Ano5 | ENSMUSG00000055489 | -1,66383 | 0,00126 | 0,01546 |
| Kcna2 | ENSMUSG00000040724 | -1,67434 | 9,02E-09 | 8,02E-07 |
| Rp1 | ENSMUSG00000025900 | -1,68179 | 0,00404 | 0,03659 |
| Ucp3 | ENSMUSG00000032942 | -1,70834 | 2,06E-07 | 1,22E-05 |
| Ankrd63 | ENSMUSG00000078137 | -1,72267 | 0,00199 | 0,02154 |
| Rasef | ENSMUSG00000043003 | -1,72808 | 0,00118 | 0,01467 |
| Shisa6 | ENSMUSG00000053930 | -1,73801 | 0,00134 | 0,0162 |
| Efemp1 | ENSMUSG00000020467 | -1,79069 | 2,01E-16 | 9,64E-14 |
| Kcnv2 | ENSMUSG00000047298 | -1,84132 | 3,01E-10 | 3,90E-08 |
| Klhl33 | ENSMUSG00000090799 | -1,8481 | 1,37E-13 | 3,75E-11 |
| Sfrp5 | ENSMUSG00000018822 | -1,89377 | 2,08E-05 | 5,84E-04 |
| Penk | ENSMUSG00000045573 | -1,93268 | 9,50E-05 | 0,00196 |
| Gm10635 | ENSMUSG00000111765 | -1,95823 | 2,89E-08 | 2,28E-06 |
| Retnla | ENSMUSG00000061100 | -1,95896 | 1,90E-12 | 4,06E-10 |
| Acot1 | ENSMUSG00000072949 | -2,02293 | 5,50E-04 | 0,00809 |
| Sbk3 | ENSMUSG00000085272 | -2,05686 | 3,03E-07 | 1,71E-05 |
| Uckl1os | ENSMUSG00000010492 | -2,0688 | 2,21E-09 | 2,34E-07 |
| Alkal2 | ENSMUSG00000054204 | -2,09591 | 9,82E-06 | 3,21E-04 |
| Inmt | ENSMUSG00000003477 | -2,22303 | 1,32E-05 | 4,03E-04 |
| Aldob | ENSMUSG00000028307 | -2,71191 | 3,96E-22 | 5,14E-19 |
| Pfkfb1 | ENSMUSG00000025271 | -2,77659 | 9,77E-20 | 7,75E-17 |
| Car3 | ENSMUSG00000027559 | -2,81698 | 2,39E-04 | 0,00406 |
| Ccl11 | ENSMUSG00000020676 | -2,87303 | 3,82E-09 | 3,87E-07 |
| Cacng6 | ENSMUSG00000078815 | -3,05245 | 2,39E-05 | 6,49E-04 |
| Aqp4 | ENSMUSG00000024411 | -3,20318 | 1,79E-04 | 0,00325 |

**Differentially expressed genes in the left ventricle (WT-TAC vs. HCN4F-TAC)**

| Gen Symbol | Gen ID | log2FoldChange | p-value | Padj |
| --- | --- | --- | --- | --- |
| Vgll2 | ENSMUSG00000049641 | 2,8013 | 5,29E-06 | 0,00244 |
| Gm29773 | ENSMUSG00000110547 | 2,43177 | 2,85E-06 | 0,00165 |
| Has1 | ENSMUSG00000003665 | 2,21234 | 5,69E-04 | 0,04764 |
| Crlf1 | ENSMUSG00000007888 | 2,11645 | 2,01E-04 | 0,02546 |
| Itih2 | ENSMUSG00000037254 | 1,99304 | 1,54E-05 | 0,00444 |
| Frem1 | ENSMUSG00000059049 | 1,94828 | 1,15E-04 | 0,01689 |
| Col8a2 | ENSMUSG00000056174 | 1,9112 | 1,93E-04 | 0,02533 |
| Serpinb1c | ENSMUSG00000079049 | 1,90622 | 5,78E-06 | 0,00246 |
| Ccl12 | ENSMUSG00000035352 | 1,87335 | 3,75E-04 | 0,03703 |
| Gdf15 | ENSMUSG00000038508 | 1,86738 | 2,16E-08 | 2,48E-05 |
| Gm13054 | ENSMUSG00000086806 | 1,77535 | 1,75E-06 | 0,00121 |
| Syndig1 | ENSMUSG00000074736 | 1,68834 | 1,46E-05 | 0,00429 |
| Etv4 | ENSMUSG00000017724 | 1,6338 | 8,73E-05 | 0,01443 |
| Atp6v0a4 | ENSMUSG00000038600 | 1,62941 | 2,51E-04 | 0,02885 |
| Grip1 | ENSMUSG00000034813 | 1,61599 | 1,31E-04 | 0,01885 |
| Ptgs2 | ENSMUSG00000032487 | 1,58665 | 1,06E-05 | 0,0035 |
| Lockd | ENSMUSG00000098318 | 1,5312 | 1,43E-04 | 0,01993 |
| Ccl2 | ENSMUSG00000035385 | 1,47876 | 1,26E-04 | 0,01837 |
| Timp1 | ENSMUSG00000001131 | 1,46867 | 8,02E-05 | 0,01386 |
| Col12a1 | ENSMUSG00000032332 | 1,46576 | 1,35E-05 | 0,00406 |
| Acta1 | ENSMUSG00000031972 | 1,41463 | 3,16E-07 | 2,55E-04 |
| Ngef | ENSMUSG00000026259 | 1,36558 | 2,25E-04 | 0,02691 |
| Thbs1 | ENSMUSG00000040152 | 1,36277 | 3,29E-04 | 0,03439 |
| Lrp8 | ENSMUSG00000028613 | 1,35986 | 3,52E-04 | 0,03549 |
| Gdf6 | ENSMUSG00000051279 | 1,35935 | 2,09E-05 | 0,00526 |
| Itih4 | ENSMUSG00000021922 | 1,30475 | 6,47E-06 | 0,00253 |
| AI593442 | ENSMUSG00000078307 | 1,26995 | 2,34E-07 | 2,02E-04 |
| Ccl7 | ENSMUSG00000035373 | 1,26531 | 1,66E-05 | 0,00445 |
| Egr3 | ENSMUSG00000033730 | 1,26333 | 9,35E-05 | 0,01505 |
| Nrg1 | ENSMUSG00000062991 | 1,25701 | 5,04E-05 | 0,00994 |
| Tbx15 | ENSMUSG00000027868 | 1,2542 | 2,34E-06 | 0,00154 |
| Nlrc3 | ENSMUSG00000049871 | 1,24252 | 3,40E-04 | 0,03459 |
| Sox9 | ENSMUSG00000000567 | 1,23417 | 3,23E-09 | 5,58E-06 |
| Frzb | ENSMUSG00000027004 | 1,22257 | 1,01E-05 | 0,00339 |
| Col8a1 | ENSMUSG00000068196 | 1,19426 | 3,55E-10 | 7,01E-07 |
| Foxs1 | ENSMUSG00000074676 | 1,13274 | 6,45E-05 | 0,01143 |
| Itgbl1 | ENSMUSG00000032925 | 1,12478 | 2,94E-06 | 0,00165 |
| Ankrd1 | ENSMUSG00000024803 | 1,10822 | 6,09E-07 | 4,43E-04 |
| Pstpip1 | ENSMUSG00000032322 | 1,08898 | 2,73E-04 | 0,02959 |
| Mfap5 | ENSMUSG00000030116 | 1,08378 | 6,05E-06 | 0,00246 |
| Aqp8 | ENSMUSG00000030762 | 1,07598 | 1,66E-05 | 0,00445 |
| Tnc | ENSMUSG00000028364 | 1,07394 | 1,99E-04 | 0,0254 |
| Edn3 | ENSMUSG00000027524 | 1,04487 | 4,40E-05 | 0,00916 |
| Vcan | ENSMUSG00000021614 | 1,02894 | 4,29E-12 | 1,19E-08 |
| Meox1 | ENSMUSG00000001493 | 1,02232 | 2,67E-05 | 0,00625 |
| Scml4 | ENSMUSG00000044770 | 1,01871 | 6,02E-06 | 0,00246 |
| Apod | ENSMUSG00000022548 | 1,00637 | 6,06E-06 | 0,00246 |
| Cenpf | ENSMUSG00000026605 | -1,01599 | 3,98E-04 | 0,03854 |
| Abca4 | ENSMUSG00000028125 | -1,0295 | 3,38E-04 | 0,03456 |
| Nnmt | ENSMUSG00000032271 | -1,03504 | 1,11E-04 | 0,01689 |
| Kcnv2 | ENSMUSG00000047298 | -1,11774 | 4,16E-04 | 0,03994 |
| Klhl33 | ENSMUSG00000090799 | -1,15522 | 6,75E-05 | 0,01181 |
| Acsm5 | ENSMUSG00000030972 | -1,24714 | 2,54E-05 | 0,00611 |
| Pts | ENSMUSG00000032067 | -1,31677 | 2,28E-29 | 1,05E-25 |
| Pfkfb1 | ENSMUSG00000025271 | -1,42027 | 2,78E-06 | 0,00165 |
| Uckl1os | ENSMUSG00000010492 | -1,47493 | 4,60E-04 | 0,04204 |
| Dixdc1 | ENSMUSG00000032064 | -1,59791 | 6,46E-15 | 2,23E-11 |
| Nxpe2 | ENSMUSG00000032028 | -1,85166 | 3,33E-07 | 2,55E-04 |
| Aqp4 | ENSMUSG00000024411 | -2,40798 | 1,82E-05 | 0,00466 |
| Nrg4 | ENSMUSG00000032311 | -2,6601 | 3,37E-10 | 7,01E-07 |
| Pdzd3 | ENSMUSG00000032105 | -2,78249 | 5,01E-06 | 0,00239 |
| Gm11942 | ENSMUSG00000094344 | -3,91622 | 6,63E-06 | 0,00253 |
| Nxpe4 | ENSMUSG00000044229 | -4,91515 | 1,29E-52 | 1,79E-48 |
| Plet1os | ENSMUSG00000101304 | -5,66574 | 1,60E-35 | 1,11E-31 |
